# SIRT6-dependent functional switch via K494 modifications of RE-1 silencing transcription factor

**DOI:** 10.1038/s41419-024-07160-0

**Published:** 2024-11-07

**Authors:** Adam Zaretsky, Alfredo Garcia Venzor, Ekaterina Eremenko, Daniel Stein, Dmitrii Smirnov, Yuval Rabuah, Rebecca Dryer, Dmitrii Kriukov, Shai Kaluski-Kopatch, Monica Einav, Ekaterina Khrameeva, Debra Toiber

**Affiliations:** 1 Department of Life Sciences, Ben-Gurion University of the Negev, Beer Sheva, 8410501 Israel; 2 The Zlotowski Center for Neuroscience, Ben-Gurion University of the Negev, Beer Sheva, 8410501 Israel; 3 Center for Molecular and Cellular Biology, Skolkovo Institute of Science and Technology, Moscow, 121205 Russia

**Keywords:** Epigenetics, Transcription

## Abstract

RE-1 silencing transcription factor (REST) is a key repressor of neural genes. REST is upregulated under stress signals, aging and neurodegenerative diseases, but although it is upregulated, its function is lost in Alzheimer’s Disease. However, why it becomes inactive remains unclear. Here, we show that the NAD-dependent deacetylase SIRT6 regulates REST expression, location and activity. In the absence of SIRT6, REST is overexpressed but mislocalized, leading to a partial loss of its activity and causing it to become toxic. SIRT6 deficiency abrogates REST and EZH2 interaction, perturbs the location of REST to the heterochromatin Lamin B ring, and leads to REST target gene overexpression. SIRT6 reintroduction or REST methyl-mimic K494M expression rescues this phenotype, while an acetyl-mimic mutant loses its function even in WT cells. Our studies define a novel regulatory switch where, depending on SIRT6 presence, the function of REST is regulated by post-translational modifications on K494 (Ac/me), affecting neuronal gene expression.

**Figure:**
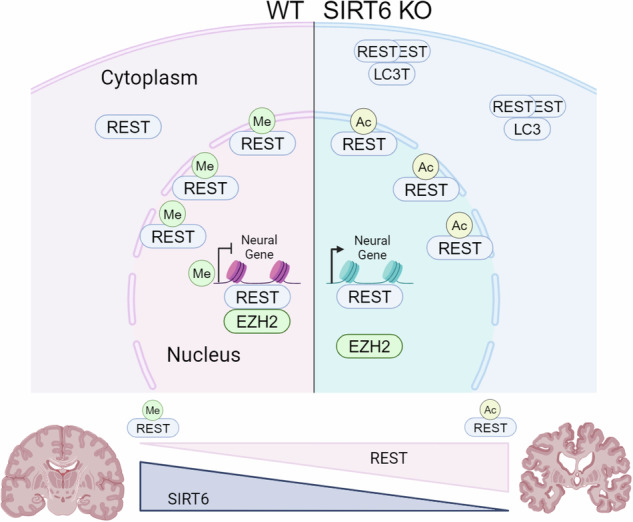
In WT cells (left), REST functions as a repressor due to its methylation, which allows proper localization and interaction with EZH2. In SIRT6 KO cells (right), REST is overexpressed, but it is mislocalized and acetylated instead of methylated, impairing its interaction with EZH2. REST localizes in the cytoplasm in autophagosomes. The overall increase in REST without SIRT6 results in non-functional and toxic REST proteins. During aging, SIRT6 declines in the brain, while REST is upregulated to protect it. In pathological aging, where SIRT6 levels are very low, the increase in REST without SIRT6 results in non-functional and toxic REST.

In WT cells (left), REST functions as a repressor due to its methylation, which allows proper localization and interaction with EZH2. In SIRT6 KO cells (right), REST is overexpressed, but it is mislocalized and acetylated instead of methylated, impairing its interaction with EZH2. REST localizes in the cytoplasm in autophagosomes. The overall increase in REST without SIRT6 results in non-functional and toxic REST proteins. During aging, SIRT6 declines in the brain, while REST is upregulated to protect it. In pathological aging, where SIRT6 levels are very low, the increase in REST without SIRT6 results in non-functional and toxic REST.

## Introduction

The RE-1 silencing transcription factor (REST) is a zinc finger DNA binding transcription factor (TF). REST is a master regulator of neural genes [1] which predominantly acts as a repressor [2]. During development, REST is a key factor for proper cell differentiation and a repressor of neural phenotype in non-neural somatic tissues [3]. REST is silenced in differentiated neurons during adulthood, but upregulated under neuronal intrinsic or extrinsic insults [4]. Interestingly, REST expression is elevated in aging brains, serving as a neuroprotective factor [5]. However, in the brain of AD patients, REST fails to localize to the nucleus, thereby failing in its neuroprotective role as a gene expression regulator [5]. Differentiated induced pluripotent stem cells (iPSC) derived from AD patient fibroblasts showed that these patients have a distinct gene expression profile, presenting upregulation in neurodevelopment and neural activity genes. Interestingly, most of these genes are REST targets, suggesting that in neurodegeneration REST target genes are misregulated; however, the impaired function of REST as a repressor remains unclear [6].

Sirtuin 6 (SIRT6), a histone deacetylase and ADP-ribosyl transferase, plays a crucial role in various cellular functions such as gene expression [7] and DNA repair [8] involved in organismal aging and neurodegeneration [7, 9–11]. “SIRT6-KO monkeys” die immediately following birth, presenting severe physiological abnormalities—specifically, impaired brain development. Moreover, in humans with a mutant SIRT6 that renders it inactive, embryonic lethality and neurodevelopmental disorders occur. On the other hand, during aging, SIRT6 diminishes, and an even more pronounced reduction is observed in AD patients. Brain-specific SIRT6-deficient mice develop a neurodegenerative-like phenotype with hyperphosphorylated and hyper-acetylated Tau, learning impairment, and increased cell death [10, 12], impaired sleep patterns [13], and loss of proteostasis [14]. It follows that both REST and SIRT6 play a role in brain development and maintenance, thus preventing neurodegeneration.

In this study, we find that SIRT6-KO brains show that a significant part of the upregulated genes are REST targets. We therefore expected REST to be downregulated. Instead, we find that in the absence of SIRT6, REST is overexpressed, as seen in AD patients. However, its repressive function is impaired, leading to an upregulation of its target genes. Although REST itself is upregulated, it is mislocalized inside the nucleus and in the cytoplasm, where it forms aggregate-like structures due to changes in post-translational modification in residue K494. In the nucleus, it cannot localize properly to the Lamin B domain, and the recruitment of EZH2 and binding to H3K27me3 is impaired. This suggests that, in the absence of SIRT6, the brain cannot be protected due to the loss of function of REST, despite its upregulation.

## Results

### SIRT6-deficient brains show impaired expression of neuronal activity genes

**Fig. 1:**
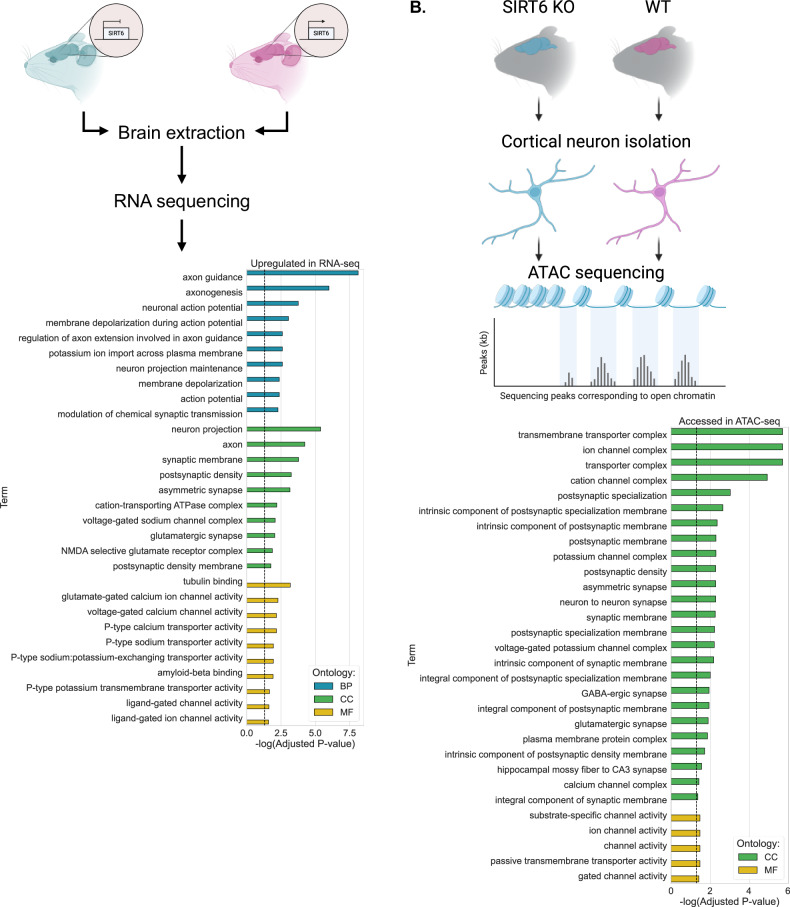
Neural genes are upregulated in brS6KO brain. **A** Enrichment analysis of upregulated genes in brS6KO mice. **B** Enrichment analysis of differentially accessed genes in SIRT6-KO cortical neurons. BP biological processes, CC cellular component, MF molecular function. Dashed line represents Adjusted *P*-value = 0.05.

To characterize the transcriptional profile of the brS6KO brain tissue, we performed whole-brain RNA sequencing on brS6KO and WT mice (Fig. S1A). Gene enrichment against several datasets of the differentially overexpressed genes showed enrichment of categories in Gene Ontology (GO) related to neural activity and development (Fig. 1A, Table S1). These results suggest that SIRT6 is involved in regulating brain function and neuronal activity. To understand the changes in cortical neurons specifically, we performed ATAC sequencing (ATAC-seq) on WT and brS6KO cortical neurons isolated from 10-month-old mice, allowing us to recognize genomic regions accessible to transcription mechanisms (Fig. S1B, S1C). Enrichment of regions differentially accessed in brS6KO neurons reveals the significant enrichment of GO categories associated with neural activity (Fig. 1B, Table S2). Genes represented in both RNA-seq and ATAC-seq were mainly related to neural activity, morphology, and development (Fig. 1A, B).

### SIRT6-KO differentially expressed genes are enriched for REST regulation

**Fig. 2:**
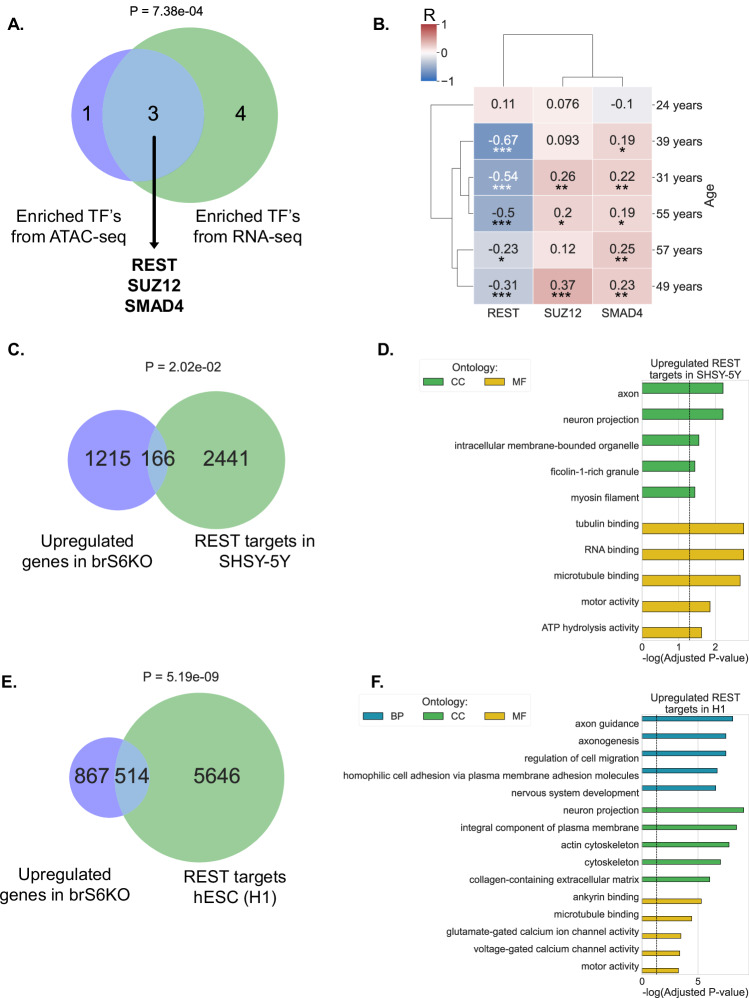
REST regulates upregulated genes in SIRT6 KO. **A** Hypergeometric test of TFs targeting differentially accessed genes in SIRTKO cortical neurons ATAC-seq, with differentially expressed genes in brS6KO RNA-seq. In both data sets SUZ12, SMAD4, and REST are common between these two enrichment analyses. **B** Hierarchical clustering of expression profile correlation between SIRT6 and each of the candidate transcription factors. n(brains) = 6. Value in each cell represents Pearson R. **C** Hypergeometric test of genes upregulated in brS6KO, and genes significantly targeted by REST SHSY-5Y ChIP-seq performed by Lu et al., 2014. **D** Genes that were common in both datasets in (**C**) were enriched against GO categories. **E** Hypergeometric test of genes upregulated in brS6KO, and genes significantly targeted by REST in human embryonic stem cells (H1) ChIP-seq (GSM803365). **F** Genes that were common in both datasets in (**E**) were enriched against GO categories. *p < 0.05, **p < 0.01, ***p < 0.001. BP biological processes, CC cellular component, MF molecular function. Dashed line represents Adjusted *P*-value = 0.05.

Next, we asked whether these genes might be regulated by a common transcription factor (TF). To find potential TF candidates, we performed an enrichment analysis of the brS6KO cortical neuron ATAC-seq and the brS6KO RNA-seq against the ENCODE, and ChEA consensus TFs from a ChIPX database. We then performed a hypergeometric meta-analysis, encompassing the enriched TFs, to find the candidates overlapping both neuron chromatin accessibility and brain expression. Our filtered results identified three potential TFs: SUZ12, SMAD4, and REST (Fig. 2A). To narrow our focus, we tested their relevance to SIRT6 expression in the human brain by comparing the transcription profile of SIRT6 from six human brains (Allen Brain Atlas) to the profile of each TF [15]. Interestingly, the RE-1 Silencing Transcription factor (REST) correlates negatively with SIRT6 and, most significantly, in the brains of older individuals. The transcription factors SMAD4 and SUZ12 showed a significant but less prominent correlation with SIRT6 in most individuals (Figs. 2B, S1A). REST is considered a repressive factor crucial for mediating the expression of neural development and activity genes containing the RE-1 element in their promoter [16]. Therefore, these results suggest that upregulated genes in brS6KO might be mediated through a lack of REST repression.

Next, we examined the proportion of REST targets among the upregulated genes in brS6KO. To obtain this result, we overlapped the SIRT6 RNA-seq results with publicly available REST ChIP-seq data of SHSY-5Y cells [5], and H1 cell line ChIP seq (GSM803365) (Fig. 2C, E). Over 100 genes upregulated in brS6KO are REST targets in the SHSY-5Y cell line, enriched in GO categories related to neural activity and development (Fig. 2D, Table S3). In H1 cells, more than 500 REST targets are represented in brS6KO upregulated genes and are associated with neural morphology and activity (Fig. 2F, Table S4). Altogether, our results suggest that the transcriptional alteration in brS6KO, specifically the upregulated neural genes, is likely mediated through REST.

### REST is overexpressed in SIRT6-KO brains and cells

**Fig. 3:**
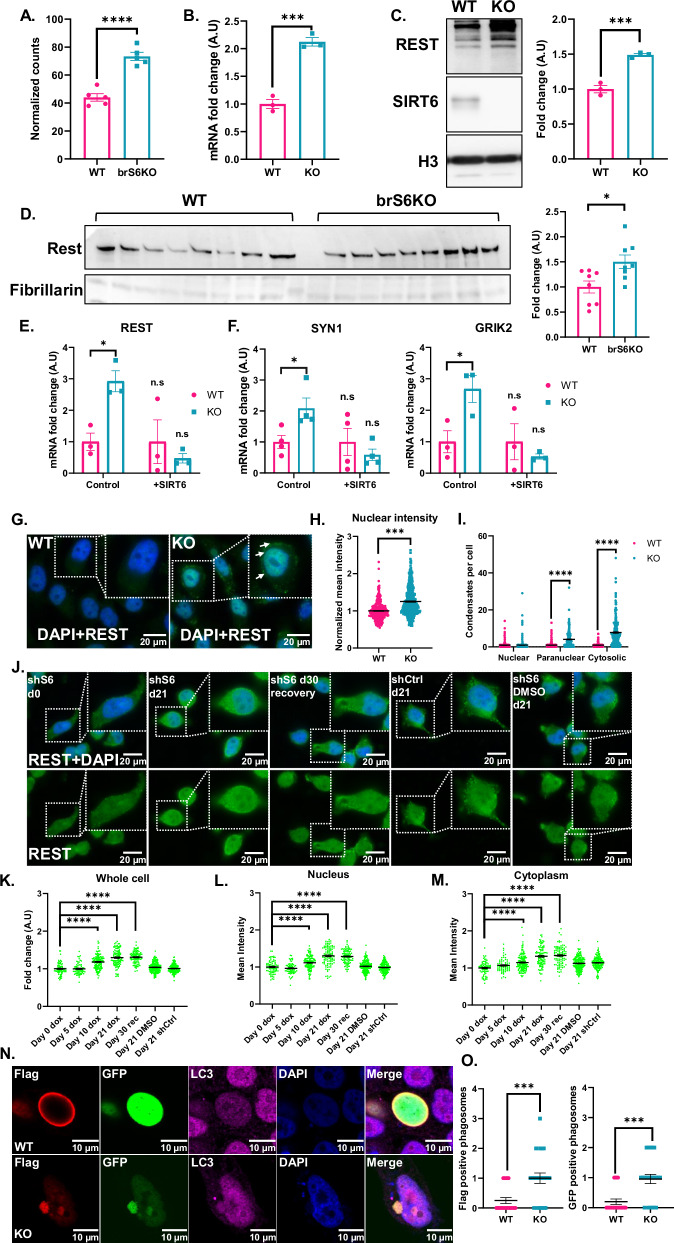
REST expression is upregulated in SIRT6 KO. **A** Normalized counts from brS6KO RNA-seq of REST, n(mice) = 5. **B** mRNA fold change via qPCR of REST in SHSY-5Y cell lines. *n* = 3. **C** Chromatin protein extraction from SHSY-5Y cell line. *n* = 3. **D** REST protein levels in chromatin extraction from WT and SIRT6 KO mice brains, *n* (mice per group) = 8. **E** mRNA fold change via qPCR of REST in WT vs KO SHSY-5Y cells transfected SIRT6 or Control vector. *n* = 3. **F** mRNA fold change via qPCR of SYN1 *n* = 4 and GRIK2 *n* = 3 in SHSY-5Y cell line. **G** Immunofluorescence of REST (Alexa flour 488) in WT and KO SHSY-5Y cells. **H** Quantification of nuclear REST intensity in WT and KO SHSY-5Y cells. n(cells) = 294[WT],369[KO]. **I** Quantification of REST condensations via ImageJ tool ”AggreCount”. n(cells) = 256. **J** Immunofluorescence of endogenous REST in SHSY-5Y cells with inducible SIRT6 silencing. shSIRT6 (shS6) Day 0 (d0) dox represent cells with shSRIT6 without silencing. shS6 d21 dox represent cells with shSIRT6 treated 21 days with dox (21 days of silencing). d30 recovery are cells that did not receive Dox since d21. shCtrl are cells induced to express scramble shRNA. DMSO is vehicle control instead of doxycycline. **K**–**M** Quantification of REST intensity in whole cell, nuclear, and cytoplasm in shSIRT6 inducible system, 21 days of shSIRT6 induction, followed by recovery (until day 30). n(cells) = 72[Day 0 dox], 66[Day 5 dox], 130[Day 10 dox], 99[Day 21 dox], 93[Day 30 rec], 179[Day 21 DMSO], 149[Day 21 shCtrl]. **N** Immunofluorescence of Flag-REST-GFP transfected WT and SIRT6 KO SHSY-5Y cells. The Flag channel is in red, GFP in green, LC3 in magenta, DAPI in blue. **O** Quantification of Flag/GFP positive phagosomes detected by cytoplasmic LC3. Each dot represents a cell. n(cells) = 20[WT],22[KO]. Bars and error bars represent Mean ± SEM. **A**–**D**, **H**, **O** Unpaired t-test. **E**–**F** Two-way ANOVA followed by Sidak’s multiple comparison. F(SYN1) Multiple t-test. **I**–**M** Two-way ANOVA followed by Sidak’s/Dunnett’s multiple comparison. Two-tailed *p < 0.05, **p < 0.01, ***p < 0.001, ****p < 0.0001.

REST is known as a neural gene repressor, and since its targets were upregulated, we expected to observe reduced levels of REST in brS6KO. Surprisingly, REST levels were higher in the brS6KO RNA-seq data (Fig. 3A). To validate these results in cells, we measured mRNA and protein levels of REST in the SHSY-5Y cell line with CRISPR deleted SIRT6 (SIRT6-KO). In these cells, REST was overexpressed in both mRNA and protein levels of chromatin extractions (Fig. 3B, C). In brS6KO mice, REST protein levels were increased in chromatin extracts (Fig. 3D).

To confirm that this overexpression is a product of SIRT6 absence, we introduced recombinant SIRT6 to the SIRT6-KO cells. mRNA measurement by qPCR shows rescue of REST levels with the introduction of SIRT6 (Fig. 3E), suggesting that the expression of this factor is SIRT6 dependent. To test whether this is a conserved phenomenon, and if we could use tissue culture to understand REST-related changes, we selected REST target genes from the RNA-seq results to measure in the SIRT6-KO cells (Fig. S3A). We measured mRNA levels for two representative REST target genes, SYN1 and GRIK2, and found that in both genes, mRNA levels were higher in SIRT6-KO cells, and that reintroduction of SIRT6 to these cells rescued their expression (Fig. 3F). In the context of AD, SIRT6 expression declines with Braak stage progression [10]. To assess the levels of REST expression in AD, we used microarray data from AD and non-AD patients from the GSE48350 database. By dividing the samples according to their Braak stage, we concluded that REST expression is higher in later Braak stages than earlier ones, suggesting loss of expression regulation (Fig S3B).

REST has several isoforms, some of which lack repressive capabilities and thus quench the function of the main isoform [17, 18]. To test for the possibility of alternative splicing, we constructed probes that recognize the exon 2 and exon 3 (E2 + E3) exon junction (present in most of the transcripts) and the exon 3 and exon 4 (E3 + E4) exon junction (present in the full isoform). We measured the abundance of the inactive splice variant relative to the whole isoform population (Fig. S3C). According to our measurements, there was no significant difference in the main REST isoform population between WT and SIRT6-KO cells (Fig. S3D), indicating that REST loss of function does not correlate with REST alternative splicing, and the lack of repressive capacity is not from the generation of different alternative spliced variants.

### REST sub-cellular localization

To address the hypothesis of REST loss of function in SIRT6 KO cells, we first tested changes in cellular localization. Previous studies showed that REST could be nuclear and cytoplasmic [19]. We hypothesized that changes in REST localization might hint at the causality of its impairment. To observe nuclear morphology and different nuclear regions, we performed immunofluorescence (IF) against the endogenous REST in SIRT6-KO cells (Fig. 3G). When measuring total cell intensity, the intensity of REST significantly increased in KO cells (Fig. 3G, H). Dividing the cellular area into its nuclear and cytoplasmic regions, we observed that REST intensity was higher in both compartments (Figs. 3H and S3E). In addition, in the SIRT6-KO cells, REST was observed as condensates in the cytoplasm and closer to the cellular membrane, in addition to its nuclear location (Fig. 3G). This phenotype was not observed in WT cells. Quantification of these bodies shows that REST condensates accumulate in the cytoplasm of the SIRT6-KO cells (Fig. 3I). To test whether this phenomenon is reversible, we used an inducible shSIRT6 system that silences SIRT6 for 21 days, using doxycycline, followed by 9 more days of recovery of SIRT6 levels (Figs. 3J and S3F). After the tenth day of silencing, REST cellular intensity increased significantly, in agreement with our previous results from SIRT6-KO cells. Interestingly, the recovery period did not reduce the mean nuclear intensity of REST, in addition to whole cell and cytoplasmic region measurements (Fig. 3J–M). Our previous results show rescue of mRNA levels of REST after 48 h of SIRT6 reintroduction (Fig. 3E). However, in the inducible experiment, the intensity of REST does not decrease after SIRT6 recovery, suggesting that the phenotype is irreversible at the protein level, at least within 10 days after SIRT6 re-expression.

To test whether this was due to high levels of REST leading to its aggregation, we overexpressed REST with Flag-REST plasmid under the CMV promoter, followed by immunofluorescence using Flag antibodies, which recognize only the exogenous protein. In this experiment, we failed to see cytoplasmic REST bodies. To understand the discrepancy between exogenous REST and endogenous staining, we cloned a Flag-REST-GFP plasmid (Fig. S4A, B), since the antibodies are targeted for the C-terminus and Flag was in the N-terminus. Overexpression of this construct showed cytoplasmic aggregates (Fig. S3G). As a recent publication showed that REST accumulated in cytoplasmic autophagosomes [5]; we measured co-localization of REST with LC3. A fraction of REST co-localized with LC3 in both WT and SIRT6-KO cells, but there was a significant increase in SIRT6-KO cells with both Flag and GFP (Fig. 3N, O).

Last, we collected brains from mice at different ages, and separated proteins into chromatin bound and cytoplasmic fractions. With age, REST tends to localize more to the cytoplasmic fractions almost two-fold, while being depleted from the chromatin bound fraction. This suggests that the overexpression of REST first leads to its increase in all fractions, while slowly accumulating in the cytoplasm with age (Fig. 3SH, I). Note that in this method we are missing the nuclear fraction, unlike that for IF. SIRT6 shows a declining trend with age, but presents some variability (Fig. 3SJ).

### REST is present at the nuclear Lamina

**Fig. 4:**
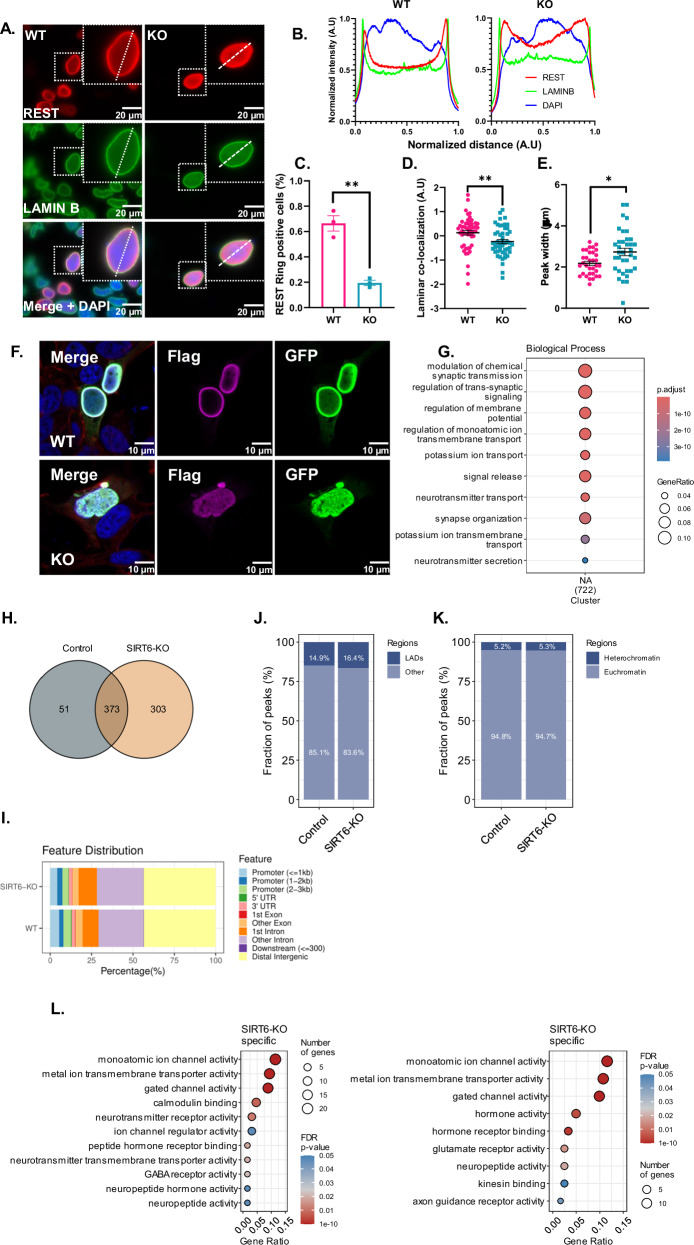
REST localization alters in SIRT6 KO. **A** Nuclear distribution of Flag-REST in SHSY-5Y WT and KO cells. Cells were immuno-stained for recombinant REST (Anti-Flag) and LAMINB as nuclear envelope reference. The dashed frame marks representative cells for quantification. **B** Representing spectra of DAPI, LAMINB, and Flag channels across the dashed line in (**A**). Distance and intensity are scaled according to the maximum value. **C** Percentage of “REST ring” positive cells out of total transfection positive cells. *n* = 3. **D** Quantification of Nuclear REST distribution. The swarm plot represents the REST nuclear distribution value per cell. n(cells) = 52[WT],50[KO]. **E** Swarm plot representing the thickness of the REST peak close to the nuclear lamina, among cells that present REST. N(cells) = 33[WT],36[KO]. **F** Immunofluorescence of Flag-REST-GFP transfected WT and SIRT6 KO SHSY-5Y cells. Flag channel in magenta, GFP in green. **G** Functional enrichment analysis of consensus REST peaks from 3 independent replicates. **H** Venn diagram representing the number of consensus peaks in WT and SIRT6 KO cells. **J** Fraction of REST peaks overlapping with laminin-associated domains (LADs) in WT vs SIRT6 KO cells. **K** Fraction of REST peaks overlapping with H3K27me3 domains. **I** Feature distribution of REST peaks in WT vs SIRT6 KO cells. **L** Go analysis of WT and SIRT6 KO REST peaks and Enrichment analysis of REST peaks specific to SIRT6 KO cells. All data represents Mean ± SEM. **C**–**E** Unpaired t-test. Two tailed *p < 0.05, **p < 0.01.

Interestingly, we detected REST accumulating closer to the nuclear lamina, forming a ring almost co-localizing with Lamin B in WT cells (Fig. 4A, B). This was not previously seen in REST. In contrast, SIRT6-KO cells exhibited less co-localization of REST with the nuclear lamina (70% less) (Fig. 4C). When a ring was detectable, it was more homogenous and thicker inside the nucleus, measured by the co-localization of REST to the nuclear lamina and the thickness of the REST peak near the lamina (Fig. 4B, D–F). This suggests a loss of heterochromatin binding, or loss of chromatin condensation capability. Moreover, Flag and GFP staining co-localize in this ring, but not in all the cells, with GFP having a more homogenous nuclear distribution, and higher appearance in the cytoplasmic fraction (Fig. S4B). These results suggested that REST could be cleaved, separating the C and N terminus. To test this possibility, we transfected Flag-REST-GFP and measured total protein extracts as well as cytoplasmic and chromatin-bound fractions with Flag, GFP, and REST antibodies. REST fragmentation is clearly visible overall (also with a higher band, only in total extraction). While Flag shows mainly a chromatin bound presence, GFP could be seen in the cytoplasm, as well as REST (which combined both exogenous and endogenous forms). This strengthens the point at which N-terminal REST is mainly nuclear and chromatin bound, while the C-terminus is cleaved and appears more in the nucleus as cytoplasm (Fig. S4F–H).

Next, we performed ChIP-seq for a clearer understanding of the changes in REST targets in SIRT6-WT and KO cells. We confirmed a general enrichment for neuronal-related genes (Fig. 4G). SIRT6 deficient cells have almost double the number of peaks, probably due to higher levels in the cells (Fig. 4H), but show no difference in the promoter, intron or exon distribution (Fig. 4I). Importantly, in both WT and KO cells, a significant part of REST localizes to Lamin Associated Domains (LADs) confirming our IF results. However, the KO cells show a slightly higher proportion in the LADs (Fig. 4J). A small but significant fraction of REST co-localized with heterochromatic mark H3K27me3 in both cells (Fig. 4K), but had zero overlap with H3K9me3 mark. REST gained categories such as neurotransmitter transport and other ion activities, but lost monoatomic ion channel activity, possibly affecting neuronal activity (Fig. 4L).

### REST interaction with EZH2 is abrogated in SIRT6-KO cells

**Fig. 5:**
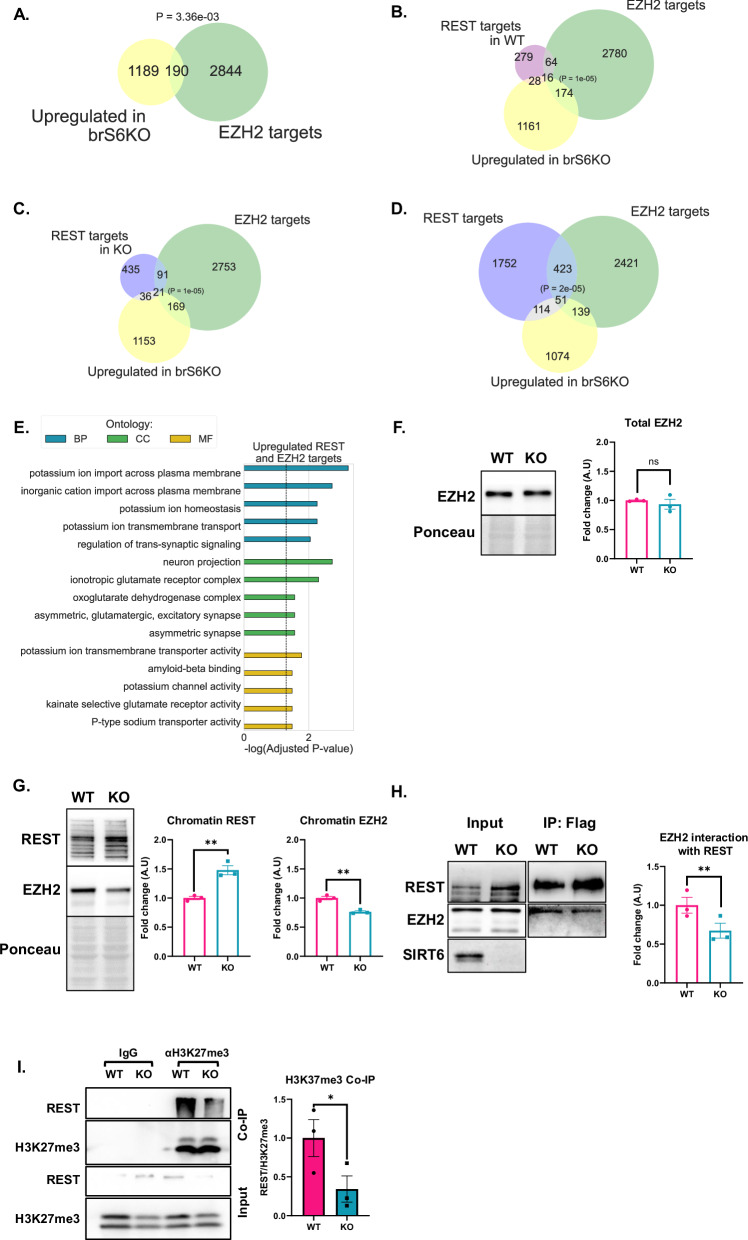
REST loses interaction with EZH2 in SIRT6 KO. **A** Venn diagram of upregulated genes in brS6KO and EZH2 hESC targets. P represents hypergeometric test *p* value. **B**, **C** Overlapping genes between REST peaks in WT (**B**) or SIRT6 KO (**C**) cells with EZH2 targets form hESC and genes upregulated in brS6KO. Calculated P resembles permutation test on the background of all available human protein-coding genes (HGNC). **D** Overlap between REST targets, EZH2 targets, and brS6KO upregulated genes. Calculated P resembles permutation test on the background of all available human protein coding genes (HGNC). **E** Enrichment analysis for overlapping genes in (**B**). **F** Total EZH2 from total protein extraction. *n* = 3. **G** Chromatin extraction of SHSY-5Y cells. *n* = 3. **H** Co-immunoprecipitation of Flag-REST. Loading normalized to equal levels of REST in the IP. n(replicates) = 3. **I** Co-immunoprecipitation of H3K27me3 in WT vs SIRT6 KO SHSY-5Y cells. Quantification represents intensities of REST/H3K27me3. *n* = 3. Bars and error bars represent Mean ± SEM. A *P* value represents hypergeometric test. **B**–**D** Permutation test *P* value. **F**–**H** Unpaired t-test. **H**, **I** Paired t-test. *p < 0.05, **p < 0.01. ns non-significant. BP biological processes, CC cellular component, MF molecular function. Dashed line represents Adjusted *P*-value = 0.05.

Since REST binding was not diminished in the SIRT6 deficient cells, we hypothesized that the REST function as a scaffold protein [17]—recruiting various chromatin remodelers to its RE-1 element in neural promoters—could be impaired by failure to repress its target genes. EZH2 is an H3K27 methyl transferase, part of the PRC2 silencing complex [20]. EZH2 is a known REST interactor and an important chromatin silencer in the brain [20, 21]. Previous works showed that the interaction of REST and EZH2 is important for repressing REST target genes [22]. Interestingly, 190 genes upregulated in brS6KO are EZH2 targets (Fig. 5A) (Using publicly available hESC ChIP-seq data). To better understand REST and EZH2 gene co-regulation in SIRT6 KO cells, we analyzed the EZH2 targets together with the REST consensus peaks from our ChIP-seq data (Fig. S5A). Functional enrichment analysis of REST and EZH2 targets revealed that overlapping pathways are neuron-related (Fig. S5B, C). We then checked if upregulated REST targets in brS6KO are also targeted by EZH2. A permutation test showed that REST and EZH2 regulate 16 genes in WT cells upregulated in brS6KO (Fig. 5B), and 21 genes in SIRT6 KO cells upregulated in brS6KO (Fig. 5C). Functional similarity between these genes allows significant enrichment in GO categories associated with neural pathways (Fig. 5E). Due to the small coverage of our ChIP-seq, we analyzed data from hESC for EZH2 and REST, and found that they overlap in about 500 genes, with 51 being also overexpressed in SIRT6-KO brains (Fig. 5D). Genes were enriched for synaptic and brain functioning, similar to the ATAC-seq and RNA-seq results (Fig. 1A, B). To confirm these bioinformatic results, we tested the levels of EZH2 in SIRT6-deficient cells. Although total levels of EZH2 were not altered in the total cell extract (Figs. 5F and S5G), the chromatin bound fractions were reduced (Figs. 5G and S5E).

To determine whether the interaction between REST and EZH2 is affected in the absence of SIRT6, we performed Co-Immunoprecipitation (Co-IP) of the recombinant Flag-REST expressed in WT vs. SIRT6 KO SHSY cells. We found a significant reduction in the interaction between REST and EZH2 in SIRT6-KO cells (Fig. 5H). Although REST did not show differential binding between WT and SIRT6 KO cells in our ChIP-seq (Fig. 4K, I), the impaired interaction with EZH2 led us to hypothesize that there are less H3K27me3 in regions bound by REST. To test this hypothesis, we performed Co-IP against H3K27me3 from chromatin preps in WT and SIRT6 KO SHSY-5Y cells, and found that REST is less enriched in H3K27me3 in SIRT6 KO cells (Fig. 5I). These results suggest that, although REST levels are increased and its DNA binding capability not impaired, interaction with key chromatin remodelers and epigenetic markers such as EZH2 and H3K27me3 are disrupted, leading to less chromatin condensation in REST bound loci.

To assess the loss and gain of REST interaction depending on SIRT6 genotype, we overexpressed recombinant REST in WT and SIRT6KO HEK293T cells and sent the triplicate samples to mass spectrometry. We obtained 141 proteins that pass the filter (excluding non-specific protein reads that were present in the empty vector sample threshold and in 3 independent IPs) (Table S5). Our results show one unique interaction for REST in WT and nine novel interactions in SIRT6-KO cells. The unique interactions were defined as proteins present specifically in the WT or KO samples (Fig. S6A). In addition, we performed a differential interaction analysis. We discovered a small but significant number of proteins [9] that are gained in SIRT6-KO cells, mainly related to translation, while the lost interactions [5] were related to DNA repair and remodeling, potentially affecting chromatin condensation function (Fig. S6B, C). STRING analysis revealed that REST interacts with proteins involved in histone and chromatin remodeling (Fig. S6D, E). For our lost and gained interactions, we performed STRING analysis and allowed the addition of a second layer of interactors to better define the functionality of these proteins (Fig S6F, G). Enrichment analysis of the cellular components of these networks revealed that, in SIRT6 KO cells, REST loses interaction with proteins involved in DNA repair, while gaining interaction with translation-related complexes—which are chiefly cytoplasmatic (Fig. S6H). These results support the fact that, in SIRT6 KO, REST exists more prominently in the cytoplasm and loses its function in the nucleus by losing key interactors. However, we cannot discard the possibility that these changes could be the result of different protein content in a SIRT6-deficient background.

### Acetylation of REST on Lysine 494 results in loss of association with the heterochromatin

**Fig. 6:**
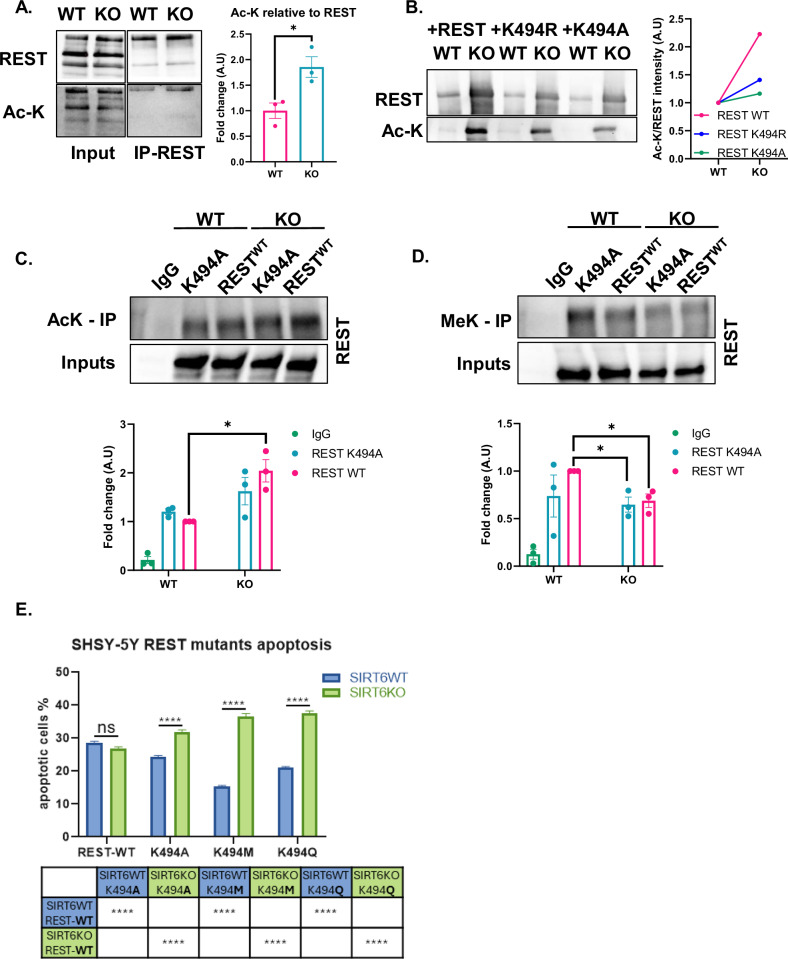
REST can be acetylated. **A** Immunoprecipitation of REST in SYSY-5Y cells transfected with Flag-REST. n(replicates) = 3. **B** Total protein extraction of SHSY-5Y cells transfected with WT REST and Arginine (K494R) or Alanine mutant (K494A). **C** Immunoprecipitation using AcK antibody. n(replicates) = 3. **D** Immunoprecipitation using Me-K antibody. n(replicates) = 3. **E** ApoAlert caspase 3 detector assay: SHSY5Y cells were co-transfected with ApoAlert and the different REST variants. The percentage of apoptotic cells was obtained per treatment and ANOVA used for analysis. Bars and error bars represent Mean ± SEM. **A** Unpaired t-test. **C** Two-way ANOVA followed by Tukey’s multiple comparisons test. **D** Unpaired t-test. *p < 0.05.

It was shown that EZH2 methylates REST on Lysine 494 (K494), increasing the interaction between these two proteins, and this modification is critical for the repression of REST target genes [22]. Since the interaction with EZH2 is diminished, we hypothesized that methylation could be impaired by the presence of different PTMs, such as acetylation. Since there are no commercially available antibodies for K494Ac, we performed immunoprecipitation (IP) of Flag-REST in WT and KO cells, and measured pan-acetyl-Lysine antibody (Fig. 6A). We discovered not only that REST is acetylated, but that it is to a greater degree in the absence of SIRT6. The reduced interaction with EZH2 and the existence of acetylated REST led us to hypothesize that the K494 residue might be responsible for these alterations. To check this finding, we generated a K494A REST mutant. Overexpression of REST variants shows that WT-REST is hyperacetylated in KO cells, while the non-modifiable K494A is generally less acetylated in WT cells and shows no change in KO cells, indicating that this is the main change occurring in SIRT6-KO cells. A similar trend is observed in the mutated K494R (Fig. 6B). These results suggest that SIRT6 mediates the balance between acetylated or methylated REST. To test the proportion of acetylated or methylated REST in SIRT6 KO cells, we performed immunoprecipitation using pan-methyl lysine (MeK) and pan-acetyl lysine (AcK) antibodies. SIRT6 KO cells transfected with WT REST showed more REST enrichment when using AcK antibody (Fig. 6C), and less when using MeK (Fig. 6D), suggesting that REST is likely to get acetylated, more than methylated, in SIRT6 KO cells. In addition, cells transfected with the non-modifiable REST K494A mutant using MeK antibody showed less enrichment of REST, regardless of the SIRT6 genotype—while in AcK, REST was enriched now similarly to the control. These results suggest that, in addition to the differential proportion of Me-REST and Ac-REST in SIRT6 KO cells, lysine 494 plays a critical role in influencing REST PTM.

To understand whether REST can become toxic under the different PTMs, we transfected the cells with the different REST variants and Apo-Alert Active caspase 3 detector. Interestingly, WT-REST was no different between SIRT6-WT and KO cells. However, the different REST variants, particularly 494 M (and to a lesser extent 494Q) could rescue the WT cells—but became more toxic in the KO cells (Fig. 6E). This suggests that in the presence of SIRT6, REST can be neuroprotective, but in its absence—as in AD—REST becomes toxic.

### REST K494 PTMs affect its nuclear location

**Fig. 7:**
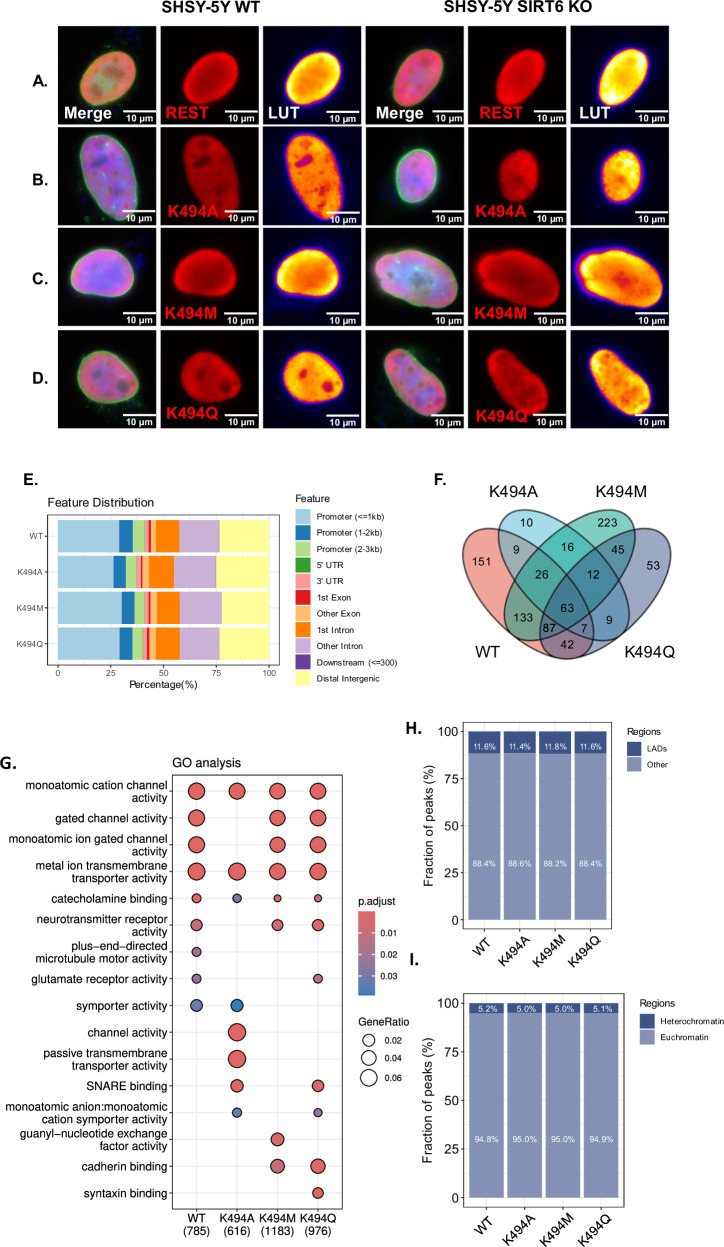
REST K494 residue influences nucellar distribution regardless of SIRT6 existence. **A**–**D** SHSY-5Y WT and SIRT6KO cells transfected with REST WT, REST K49A, REST K494M, REST K49Q, respectively. LUT editing allows to observe a heat map accumulation of REST in the nucleus. **E** Feature frequency for each mutant REST in 3 replicates. **F** Venn diagram of all peaks for 3 replicates for each REST mutant. **G** GO enrichment analysis for 2 out of 3 replicates for peaks of REST WT, K494A, K494M, and K494Q. **H** Fraction of REST peaks overlapping with H3K27me3 domains. **I** Fraction of REST peaks overlapping with laminin-associated domains (LADs).

To assess whether K494 affects REST nuclear localization, we mutated WT REST in K494 to alanine (K494A), methionine (K494M) and glutamine (K494Q). K494M and K494Q serve as PTM mimics for methylation and acetylation, respectively, in this lysine residue, while alanine is a non-modifiable mutation. REST WT and the point mutants were transfected to WT and SIRT6 KO SHSY-5Y cells (Figs. 7A–D, S7A). WT, K494A, K494M, and K494Q REST appear in the nucleus 48 h after transfection. As previously shown, WT REST co-localizes closely to the nuclear lamina in WT cells, while a more diffuse pattern is seen in SIRT6-KO cells, losing the ring feature (Fig. 7A). Interestingly, K494A REST fails to localize to the nuclear lamina in both WT and SIRTKO cells (Fig. 7B). The methyl mimic K494M showed a stronger association with the nuclear periphery even in KO cells (Fig. 7C), while the acetyl mimic showed a weaker connection even in SIRT6-WT cells (Fig. 7D). This indicates that K494 methylation induces its lamin-associated location, while non-modifiable or acetylated REST shows impaired recruitment to the nuclear envelope. In addition, we measured the width of the peaks generated by the REST mutants. SIRT6 genotype did not affect the width of the K494A and K494Q mutant peaks, probably because of the decreased accumulation of these mutants to the nuclear lamina (Fig. S7C, D). In the K494M mutant, the width of the REST peak was wider in KO than in WT cells (Fig. 7B), although the laminar colocalization value improved in the KO cells. This might suggest that, although REST is more localized to the nuclear lamina when methylated, it still depends on the presence of SIRT6 to develop a sharper localization, probably through other effects of SIRT6 on nuclear lamina function.

To understand the changes in binding activity, we performed ChIP-seq using anti-Flag antibody expressed REST WT, and the K494A, M and Q mutants in SHSY cells. Enrichment analysis of consensus peaks between all the mutants showed high enrichment of processes and pathways associated with neural activity (Fig. 7E, F). Once again, the different REST mutants did not affect the distribution of REST to the different regions such as promoter, introns, exon etc., similarly to what we saw with endogenous REST in SIRT6 WT and KO cells (Fig. 7E). We performed an analysis with 2 out of 3 ChIPs presenting the peak, and a more stringent test with the 3 peaks present. Both analyses show the same trend (Fig. S7D–F). Interestingly, the K494M mutation bound ~4 times more unique targets than K494Q, and ~22 times more than K494A. In addition, K494M shares more targets with REST WT than with K494Q (~3 times) and K494A (~8 times) (Fig. S7D). These results suggest that under normal conditions REST is methylated in the cells, allowing improved binding, while non-modifiable or acetyl mimics show an impairment.

The difference between the point mutants is mainly in the number of sites they can bind, but enrichment for the different categories is also affected. For example, WT binds the monoatomic channels, neurotransmitter and synaptic activity. Interestingly, only WT and acetyl mimic bind glutamate receptor activity, one of the enriched categories in the differentially expressed genes in brS6KO—particularly the alanine mutant loss and monoatomic ion gated channel activity. Methyl mimic lost some categories related to glutamate receptor, symporter and general transmembrane transport, but gained guanyl nucleotide exchange factor activities. One unique acetyl mimic category was syntaxin binding, also related to the SNARE binding enriched in the Alanine mutant, suggesting an overall important role for REST in membrane constitution—from ion channels to membrane composition and endocytosis (Fig. 7G).

Moreover, we see that the proportion of REST in LADs is conserved among the mutants, strengthening the notion that the plasmids are indeed binding the correct sequences as the endogenous REST, and that it is not an artifact of the Flag (Fig. 7H).

Rockowitz et al. [23] showed that REST is mainly associated with the transient heterochromatin mark H3K27me3 in neurons, and less with the more permanent H3K9me3 mark. We observe similar results here, with significant enrichment despite smaller numbers (Fig. 7I).

The association of REST K494M with the nuclear periphery led us to hypothesize that this mutant might help to repress REST target genes, even in the absence of SIRT6. To check this possibility, we introduced WT or K494M REST to normal or deficient SIRT6 cells and measured the mRNA levels of REST targets SYN1 and GRIK2 (Fig. S7G). As previously shown, SIRT6 KO influences the expression of REST targets even when overexpressed in its WT form. However, REST K494M rescues the level of these target genes, even in the context of SIRT6 deficiency. These results suggest not only that the methylation of REST is important for its repressive function, but also that SIRT6 regulates REST function through the K494 residue acetylation/methylation switch.

## Discussion

Our results indicate that SIRT6 regulates REST in multiple layers, from mRNA levels to the nucleus and cytoplasm, and represses activity at target genes. We show that REST loses its capacity to regulate gene expression through a lack of interaction with the repressor EZH2—depending on modifications in residue 494—impairing not only gene expression, but also nuclear and cytoplasmic distribution. Moreover, REST accumulates and forms cytoplasmic bodies that could become irreversibly toxic.

### SIRT6-REST neuronal targets overlap

The upregulation of neural genes in brS6KO led us to suspect misregulation through key TFs. A considerable number of genes that were upregulated in SIRT6-KO brains were found as targets of REST, in addition to other components of the PRC2 complex, like SUZ12 and EZH2. Our results showed that both REST and SUZ12 are enriched factors targeting upregulated genes in brS6KO. In cortical neurons, particularly, we could detect changes in chromatin accessibility to REST targets in SIRT6-KO brains. Absence of SIRT6 results in a dramatic transcriptomic alteration that pushes cells toward neural differentiation and unregulated neural activity, as in the phenotype of loss of REST [24]. This is similar to the iPSCs from AD patients, which experience faster differentiation to neuronal progenitor cells and later to neurons [6]. In aging brain tissue, REST represses the neural activity and development required to reduce neurotoxicity and maintain the remaining progenitor cells. Unregulated REST can lead to increased neural activity and diminish the progenitor cell population, leading to higher tissue vulnerability [6, 25]. We suggest that SIRT6 reduction during aging can misregulate REST targets in the brain, inhibiting its protective function.

### ExceREST in SIRT6-KO and aging

Our initial hypothesis was that REST levels were low in SIRT6-KO cells compared to those seen in healthy aging, where REST was found to be overexpressed and served as a neuroprotective factor [5]. Loss of REST could explain the deficit in the repression of its target genes. Surprisingly, REST is overexpressed in AD, brS6KO, and SIRT6-KO cells (qPCR, RNA-seq, and protein levels), but so are its target genes. To understand the relevance of REST in the brain, we analyzed REST and SIRT6 expression in human brains (Allen Brain Atlas) and found a significant negative correlation between the levels of SIRT6 and REST, particularly in samples of older patients. The decreased levels of SIRT6, even more pronounced in the brains of AD patients [10], could influence the levels of REST in aging brains. In the absence of SIRT6, REST fails to protect the brain.

### REST- “full gas in neutral”

In normal aging brains, REST overexpression is protective, while in AD [5] and sporadic AD cells [6], REST target genes were overexpressed due to a lack of REST localization to the nucleus. This impairment was also relevant in Parkinson’s disease [5, 6, 26]. We showed that REST was overexpressed in SIRT6-KO brains and cells; however, it was inactive, similar to what is observed in patients with AD and Parkinson’s. Since SIRT6 is reduced in aging brains and in AD, there could be a causal link. In some isoforms, REST loses its repressive domains but retains the DNA binding domain [18]. This can lead to a dominant negative phenotype, where REST isoform (REST4) competes with the full-length REST in target promoter binding. In our results, we found no difference between the main REST isoform and the total population of possible isoforms, leading us to the conclusion that regulation at the protein level was required.

### REST is mislocalized in the cytoplasm

To gain a better understanding of REST loss of function we conducted tests to determine whether it could be mislocalized, as observed in AD and PD patients. First, we observed endogenous REST protein localization in the SIRT6-KO cells. In the absence of SIRT6, REST was found in the nucleus but also showed an increase in the cytoplasm, in polarized and “condensate-like” forms. Interestingly, these bodies accumulate irreversibly and are partially co-localized in autophagosomes. While re-introduction of SIRT6 can reduce REST mRNA levels in 48 h (Fig. 3F), re-expression of SIRT6 for 10 days could not reverse this phenotype, suggesting that the loss of SIRT6 for even a short period could lead to an irreversible phenotype regarding REST protein.

### REST is mislocalized in the nucleus

In SIRT6 deficient cells, REST is highly enriched in the nucleus in a more homogeneous location, and in 70% of the cases fails to localize in a LaminB region. Our ChIP-seq results suggest that REST binding is not reduced, nor the location impaired. Therefore, its lack of function could not be explained by lack of binding. REST repressive function results from recruiting chromatin remodeling enzymes that condense histones around REST targets such as EZH2 [2]. EZH2 is a H3K27 methyl transferase and a component of the PRC2 complex [20, 22]. In the mouse cortex, loss of EZH2 leads to cortical progenitor cell depletion from the Sub Ventricular Zone and enrichment of genes related to neural development [25]. In SIRT6-KO cells, EZH2 is less abundant in chromatin. In addition, in SIRT6-KO cells the interaction between EZH2 and REST is impaired, suggesting a reason for the failure to repress its target genes. Moreover, REST usually interacts with proteins that help chromatin condensation. Our Co-IP of REST, and the loss of interaction found in SIRT6c cells by mass spec, suggest loss of chromatin remodelers and heterochromatin formation. These results might explain the appearance of a thicker REST ‘ring’ in KO cells (Fig. 4H, I), since REST still arrives to chromatin (Figs. 3C and 5A). Interestingly, during aging, the nuclear envelope is impaired, and loss of heterochromatin is observed, altering gene expression [27, 28]. Loss of REST function at LADs could contribute to this phenomenon [29–31].

### REST location and function can be controlled by modification at residue K494

EZH2 can methylate REST in a specific lysine residue (K494), which increases its interaction with EZH2. We hypothesized that this residue is also acetylated. Our results show that REST can indeed be acetylated and, interestingly, to a greater degree in SIRT6-KO cells. This might indicate a PTM balance that can influence REST function. If methylation of K494 is beneficial for REST function and interaction with EZH2, an acetylation event can keep this residue from being methylated.

When the K494 residue is mutated to alanine, REST acetylation is reduced; moreover, the acetylation increase seen in KO cells is lost, suggesting that SIRT6 could be the deacetylase. However, we failed to observe a direct interaction between SIRT6 and REST by Co-IP or mass spec (although substrate-enzyme interactions occur at a rapid rate and may be undetected in our system). Because of the lack of specific antibodies, we could not effectively measure deacetylation in vitro, and this question remains open.

REST nuclear distribution is lost when K494 is changed to alanine as a preventative for charged modifications and Q (mimicking acetylation), in both WT- and SIRT6-deficient cells. Moreover, the M mutant, which mimics methylation, pushes REST to its “functional” phenotypes even in the SIRT6-KO cells. Moreover, REST-494M can restore the gene expression of REST target genes in SIRT6-deficient cells, while WT-REST cannot, indicating that location and function can be interconnected.

All the REST PTMs mutants maintain a similar distribution in the genome (regarding regions they prefer to bind). However, the methyl-mimic can bind to a larger number of target genes than the acetyl or the non-modifiable mutants, strengthening the point that methylation helps REST in its repressive function.

Overall, we found that SIRT6 regulates the expression and activity of REST in the cell, allowing the repression of neuronal genes. This is accomplished through the use of a molecular switch in the acetylation/methylation status of REST, allowing it to interact with EZH2 and localize to nuclear lamina. The functional relevance of SIRT6 depletion in the aging brain and neurodegenerative diseases could influence the capacity of REST to protect the brain from neurodegeneration. This could be the cause of the loss of function of REST even when expressed at high levels, as observed in AD and PD; therefore, the SIRT6-REST-EZH2 pathway could be a relevant target for future research.

## Experimental model and study participant details

### Mice

Mouse models were WT C57BL6 mice (Jackson Laboratories) and WT (Cre-) and brSIRT6 KO mice developed in Dr. Toiber’s Lab.

### Cell lines

Cell lines used in the study are described in the Key Resources table. All cell lines were cultured and maintained at 37 °C, 5% CO_2_ incubation. Dulbecco’s modified Eagle medium (DMEM) (#Cat: 11965092, Gibco^TM^) supplemented with 5% L-glutamine (#Cat: A2916801, Gibco^TM^), 5% Penicillin-Streptomycin (#Cat: 15140122, Gibco^TM^) and 10% heat inactivated Fetal Bovine Serum (#Cat: 10082147, Gibco^TM^).

### Generation of brS6KO mice

SIRT6 KO mice used of this study were generated previously by Kaluski et al. [10]. In brief, Sirt6 conditional vector was inserted in a Neo cassette (flanked by two Frt seq) together with Sirt6 exon2 flanked by two loxP sites. Targeted ES cells (V6.5) were injected into C57BL6/J blastocysts. The Neo cassette was deleted in vivo by crossing the chimeras with a mouse expressing the Flpe endonuclease. Mice were backcrossed for 3 generations with C57BL6/J mice to obtain heterozygous mice that were 97% C57BL6/J background. These mice were bred with C57BL/Nestin-Cre/J mice (Jackson Laboratories).

### RNA-seq data analysis

The RNA-seq data was used from a previously published work (GSE22107) [32]. For this analysis, mice only under normal diet (ND) were analyzed. Analysis was done based on the aligned reads of 5 WT and 5 brS6KO mice. RNA-seq differential expression (DE) analysis was performed using DESeq2 [33] R package as described in previous work [32]. Differentially expressed genes were defined “Upregulated” if adjusted *P*-value was lower than 0.05 and had log2(Fold Change) > 1.5. Differentially expressed genes from RNA-seq data were enriched for functional gene categories using Enrichr function from GSEApy [34] python package. GO enrichment analysis was done against Biological processes, Cellular component, and Molecular function datasets. Transcription factor analysis was done against ENCODE and ChEA Consensus TFs from ChIP-X database.

### ATAC-seq of cortical neurons

Cortical neuron isolation and ATAC sequencing was performed according to the protocol of Eremenko et al. [35]. Three samples of ATAC-seq data were prepared per each genotype. Nextflow atacseq pipeline was for reads assembly. Differential peak analysis was conducted using Deseq2 with a particular summit interval. A peak was recognized as presented within a particular peak-summit interval if it can be found in at least 2 of 3 samples of a particular genotype. On the contrary, a peak was recognized as absent within a particular peak-summit interval if it can be found in less than 2 samples of a particular genotype. The obtained groups of emerging and vanishing peaks were annotated using R package annotatr [36]. Random peak intervals were retrieved using function randomizeRegions multiple times (random seeds are different). We retrieved randomized regions 10 times to construct an expected distribution of peak counts within a particular annotation. Assuming that the observed value of peak counts has the same standard deviation as the expected one, one-sided z-test was applied to test if the observed value significantly differs from the expected one. Differentially accessed genes from ATAC-seq data were analyzed using clusterProfiler [37] R package.

### Analysis of public REST and EZH2 ChIP-seq data

Processed data of REST ChIP-seq data from SHSY-5Y cells was used from previous published work [5]. Processed data of two human REST ChIP-seq replicated in human embryonic stem cells (hESC) (SRX3010118 and SRX3010119 accession numbers [38]) and two EZH2 ChIP-seq replicates embryonic stem cells (hESC) (SRX10382398 and SRX10382440 accession numbers [39]) were downloaded from ChIP-Atlas database [40]. Called peaks with q < 1 × 10^−5^ were annotated by their genome position using ‘annotatePeak’ function from ChIPseeker R package [41] and only peaks localized between −3000 and 3000 bp around gene transcription start site (TSS) in all replicates were analyzed. The KEGG and Biological Process GO analysis of genes associated with the selected REST and EZH2 peaks was performed using ClusterProfiler. Enrichment analysis of overlapping genes was conducted using the enrichr function from GSEApy [34] python package.

### Public human brain microarray data

Transcription profile of SIRT6, SMAD4, SUZ12, and REST from 6 human brains was downloaded from Allen Brian Atlas – human microarray database [15].

### Generation of SIRT6 KO cells

The SIRT6 KO SHSY-5Y cell line used was generated by Kaluski et al. [10]. Briefly, SH-SY-5Y cells were infected with lentivirus GeCKO system. We used 2sgRNA targeting Sirt6 CRISPR2 GCTGTCGCCGTACGCGGACA and CRISPR3 GCTCCACGGGAACATGTTTG and empty shRNA as a control. Constructions were kindly donated by the Aharoni Lab, Weizmann Institute of Science. Cells were selected by 2ug/ml puro for a week, followed by serial dilutions to single cell colony.

### RNA extraction

RNA extraction from SHSY-5Y WT and SIRT6-KO cells was performed using the EZ-RNA II total RNA isolation kit from Biological Industries according to manufacturer’s protocol.

### cDNA production

Isolated RNA (1 µg per sample) was reverse transcribed to cDNA using qScript cDNA Synthesis Kit from Quantabio, according to manufacturer’s protocol.

### qPCR

Quantitative qPCR was performed using ROCHE LightCycler® 480Probes Master or Bio-Rad SsoAdvanced Universal SYBR® Green Supermix, according to manufacturer’s protocol. qPCR data analysis was performed using −ΔΔCt method. Beta-Actin was used as housekeeping gene.

### Chromatin extraction

Cells were collected and washed in PBS and resuspended in 2–5 pellet volumes of lysis buffer (10 mM HEPES pH 7.4, 10 mM KCl, 0.05% NP-40 and protease, deacetylase and phosphatase inhibitors). For tissue samples, brains were homogenized with lysis buffer in a bullet homogenizer. Samples were incubated 20 min on ice and centrifuged at 14,000 rpm at 4 °C for 10 min. The supernatants containing the cytoplasmic proteins were removed and kept in a separate tube. Cell pellets were resuspended with 2–5 volumes of 0.2 N HCl and incubated 20 min on ice, then centrifuged at 14,000 rpm at 4 °C for 10 min. Supernatants were neutralized with an equal volume of 1 M Tris-HCl pH 8.

### Total protein extraction

For total protein extraction, cells were harvested via scraping and lysed with 1 ml of lysis buffer (KCl 150 mM, Tris HCl pH 7.5 25 mM, Glycerol 5%, Triton 0.1%, EDTA 0.2 mM, PMSF 0.2 mM, DDT 1 mM, phosphatase inhibitor 1X), incubated on ice for 30 min, then centrifuged 14,000 rpm 4 °C 30 min. Supernatant was collected.

### Western blots and immunostaining

Western blot was performed by loading protein samples (samples prepared with 4X Lameli and β-mercaptoethanol) to 6–12% acryl amid gels. After electrophoresis, proteins were transferred to a nitrocellulose membrane. Prior to immunostaining, membranes were blocked using 5% skim milk or 5% BSA. After blocking, membranes were incubated with primary antibody (1:1000) overnight. Membranes were washed 10 min and 3 times in TTBS 1X. Secondary antibody (1:10,000) was applied for 1 h in room temperature. Three more washes were conducted before membrane was revealed.

### DNA transfection

DNA transfection of recombinant DNA to cell culture was performed according to PolyJet™ DNA In Vitro Transfection Reagent manufacturer’s protocol. Cells were transfected with recombinant DNA (REST-WT, K494A, K494M, K494Q, SIRT6, CMV-Flag) 48 h prior to sample collection.

### REST expression in AD patients

REST expression values in AD patients were obtained from AD microarray data (GSE48350) [42–47]. Data was separated according to Braak stage.

### ChIP-qPCR

ChIP–qPCR analysis ChIP-seq sample preparation was carried out as previously described [48]. Briefly, SHSY-5Y cells WT and SIRT6 KO, or WT and SIRT KO transfected with Flag-REST, K494A, K494M, K494Q were cultured up to 80% of confluence. In the case of ChIP negative controls cells were transfected with an empty vector (CMV-Flag). Cells were cross-linked with 1% formaldehyde for 10 min and blocked with 0.125 M Glycine for 5 min. Cross-linked cells were scraped on RIPA buffer (150 mM NaCl, 1% NP-40, 0.5% Sodium deoxycholate, 0.1% SDS, 50 mM Tris pH 8, 5 mM EDTA, 0.5 mM PMSF, 50 mM NaF/0.2 mM Sodium orthovanadate, 5 μM trichostatin A), and chromatin was sonicated with a needle sonicator (VibraCell VCX130, Sonics, CT, USA) during 25 min, 30/30 s on/off cycles at 40% of amplitude. One milligram of the sonicated chromatin was incubated overnight with previously blocked SureBeads™ Protein G Magnetic Beads or Anti-FLAG® M2 Magnetic Beads. The beads were then washed 4 times with RIPA buffer, 4 times with LiCl buffer (500 mM LiCl, 100 mM Tris-HCl pH 8.5, 1% NP-40, 1% sodium deoxycholate), and 2 times with TE buffer (10 mM Tris-HCl pH 8, 1 mM EDTA). Chromatin was eluted from beads using elution buffer (70 mM Tris-HCl pH 8, 1 mM EDTA, 1.5% SDS) and cross-linking was reversed by adding 200 mM NaCl, 1U of Proteinase K, and incubating the eluted chromatin at 65 °C for 5 h. The obtained DNA was further purified using the Nucleo Spin Gel and PCR Clean-Up kit. For qPCR analysis of specific REST binding regions in the genome, we used the set of primers listed in the key resources table. qPCR was performed according to the method described. We used 1 μL of Input and ChIP samples per qPCR reaction.

### ChIP-seq

Endogenous REST and transfected REST samples were obtained using the same protocol for generating ChIP-qPCR samples. Eluted DNA was sent for sequencing and analysis at Azenta Life Sciences.

### ChIP-Seq library preparation and sequencing

ChIP DNA samples were quantified using Qubit 2.0 Fluorometer (Life Technologies, Carlsbad, CA, USA) and the DNA integrity was checked with 4200 TapeStation (Agilent Technologies, Palo Alto, CA, USA). ChIP-Seq library preparation and sequencing reactions were conducted at GENEWIZ, Inc/Azenta US, Inc. (South Plainfield, NJ, USA). NEB Next Ultra II DNA Library Preparation kit was used following the manufacturer’s recommendations (Illumina, San Diego, CA, USA). Briefly, the ChIP DNA was end repaired and adapters were ligated after adenylation of the 3’ends. Adapter-ligated DNA was size selected, followed by clean up, and limited cycle PCR enrichment. The ChIP library was validated using Agilent TapeStation and quantified using Qubit 2.0 Fluorometer as well as real-time PCR (Applied Biosystems, Carlsbad, CA, USA). The sequencing libraries were multiplexed and clustered on one lane of a flowcell. After clustering, the flowcell was loaded on the Illumina Novaseq instrument according to manufacturer’s instructions (Illumina, San Diego, CA, USA). Sequencing was performed using a 2 × 150 Paired End (PE) configuration. Image analysis and base calling were conducted by the Novaseq Control Software (NCS). Raw sequence data (.bcl files) generated from Illumina instrument was converted into fastq files and demultiplexed using Illumina’s bcl2fastq 2.17 software. One mismatch was allowed for index sequence identification.

### ChIP-seq data analysis in SHSY-5Y cells

Raw fastq files from both experiments were analyzed with nf-core/chipseq pipeline (v2.0) [49]. In brief, reads were trimmed with TrimGalore! [50] and align to the hg38 reference genome using BWA algorithm [51]. The peak calling step was performed with MACS2 software [52], followed by the identification of the consensus and condition-specific peak sets. Peak annotation and enrichment analysis were performed with clusterProfiler R package [53].

Publicly available Lamin B ChIP-seq human brain data [54] was used to annotate LAD regions. Heterochromatin regions in SK-N-SH cells, defined using H3K27me3 and H3K9me3 peaks, were downloaded from The Human Heterochromatin Chromatin Database (HHCDB) [55]. The significance of the overlap between peak sets used as a measure for REST occurrence at LAD or heterochromatin regions was calculated with regioneR package [56] with randomizeRegions argument in permTest() function, aiming to preserve the size of tested regions for permutations (*N* = 3000).

### Immunofluorescence

SH-SY5Y cells were washed with PBS and fixed with 4% paraformaldehyde for 10 min at RT, followed by two additional washes. Cells were permeabilized (0.1% NaCitrate, 0.1% Triton X-100, pH 6, in DDW) for 5 min and washed again. After 30 min blocking (0.5% BSA, 5% goat serum, 0.1% Tween-20 in PBS), cells were incubated with primary antibody (REST-1:1000, LAMINB-1:600, EZH2-1:500, Flag-1:900) diluted in blocking buffer overnight at 4 °C. The next day, cells were washed three times with wash buffer (0.25% BSA, 0.1% Tween-20 in PBS), incubated for 1 h with secondary antibody (diluted in blocking buffer 1:200) at RT and washed three more times. Cells were then DAPI-stained for 3 min at RT and washed with PBS twice before imaging.

### Aggregate quantification using “AggreCount” tool

REST aggregates were quantified using ImageJ plugin “AggreCount” [57]. The parameters used for aggregate quantification are: Lower 15000.000. Upper 65535.000. perinuclear distance 10.000. aggresome size 4.000. minimum aggregate size 0.200. maximum aggregate size 20.000. minimum nuclei size 40.000. nuclei strictness 10.000. minimum cell size 75.000. cell strictness 10.000. nuclei channel 2.000. nuclei stack 1.000. nuclei frame1.000. aggregate channel 1.000. aggregate stack 1.000. aggregate frame 1.000. cell channel 1.000. cell stack 1.000. cell frame 1.000. Find cells? 1.000. Segmentation? NaN. Batch mode 1.000.

### REST “ring” positive cell quantification

Immunofluorescence was performed on SHSY-5Y cells (WT and KO) transfected with recombinant Flag-REST. Cells positive to the transfection were qualitatively evaluated for the appearance of the “REST ring” appearance, where ring positive cells scored with 1, and ring negative cells scored with 0. Percentage of “REST ring” cells was determined as the proportion of ring positive cell to the number of the total transfected cells.

### REST ring quantification

Immunofluorescence was performed on SHSY-5Y cells (WT and KO) transfected with recombinant Flag-REST. Transfected REST was identified using Anti-Flag primary antibody. Anti-LAMINB primary antibody and DAPI staining were used as standard signals for laminar and homogenous signal distribution, respectively. For each Flag-REST positive cell cross section, a signal distribution plot was measured for each channel, i.e., Flag (REST), LAMINB, and DAPI. The intensity values along the cross section and length value of the cross-section were normalized to a unit value of 1 to remove intensity and length dimensions. For each channel, an area under the graph was calculated. REST Laminar co-localization value was measured as:

$${{\rm{Laminar}}}\,{\rm{co-localization}}={\log }_{10}\left(\frac{|{\rm{red}}\,{\rm{area}}-{\rm{blue}}\,{\rm{area}}|}{|{\rm{red}}\,{\rm{area}}-{\rm{green}}\,{\rm{area}}|}\right)$$

while “red area” represents Flag-REST signal area value, “green area” represents Lamin B signal area value, and “blue area” represents DAPI signal area value. The closer REST localizes to Lamin B, the higher the REST Laminar co-localization value.

### shSIRT6 cell treatment

shSRIT6 and shCtrl (scramble) cells were treated for 21, 10, 5, and 0 days with targeting SIRT6, and an empty shRNA as a control. Cells were selected by 2 μg/mL puromycin for a week.

### Immunoprecipitation

Flag tagged REST was transfected to SHSY-5Y WT and KO cells. After 48 h transfection cells were collected, harvested in 1 ml Lysis buffer (KCl 0.5 M, Tris HCl ph 7.5 50 mM, NP40 1%, DTT 0.5 mM, PMSF 0.2 mM, phosphatase inhibitor 1X) and incubated on ice for 30 min, followed by centrifugation 14,000 rpm, 30 min, 4 °C. Protein samples were normalized to equal protein quantity, and incubated with M2 - Flag magnetic beads (Sigma M8823) for 2 h in rotation, 4 °C. Beads were washed 3 times in Lysis buffer, followed by 1 wash in SDAC buffer (Tris HCl pH 9 50 mM, MgCl_2_ 4 mM, NaCl 50 mM, DTT 0.5 mM, PMSF 0.2 mM, phosphatase inhibitor 1X). Flag-bound proteins were eluted using Flag peptide for 1 h in rotation, 4 °C.

### Co-immunoprecipitation

Flag-tagged REST was transfected to SHSY-5Y WT and KO cells. After 48 h transfection cells were collected, harvested in 1 ml Lysis buffer (KCl 150 mM, Tris HCl pH 7.5 25 mM, Glycerol 5%, Triton 0.1%, EDTA 0.2 mM, PMSF 0.2 mM, DDT 1 mM, phosphatase inhibitor 1X) and incubated on ice for 30 min, followed by centrifugation 14,000 rpm, 30 min, 4 °C. Protein samples were normalized to equal protein quantity, and incubated with M2 - Flag magnetic beads (Sigma M8823) for 2 h in rotation, 4 °C. Beads were washed 2 times with Lysis buffer (KCl 150 mM), followed by 2 washes of Lysis buffer (KCl 300 mM) and one wash of Lysis buffer (KCl 150 mM). Flag-bound proteins were eluted using Flag peptide for 1 h in rotation, 4 °C.

### REST IP mass spectrometry

For preparation of REST samples for mass spectrometry, Flag-REST was overexpressed in WT and SIRT6 KO HEK293T cells. Flag-REST was immuno-precipitated according to the protocol previously described. IP samples were separated by SDS gel electrophoresis. Samples were sent for mass spectrometry and analysis at the Smoler Proteomics Center at the Technion. Proteomics results were analyzed using R, code provided.

### Quantification and statistical analysis

Pearson correlation test was performed using corr and personr functions in pandas [58] and SciPy [59] python packages, respectively. Hypergeometric test for 2 group Venn diagrams was performed using hypergeom.pmf function in SciPy python package. Permutation test for 3 group Venn diagram was performed by calculating the probability of randomly overlapping 3 groups of genes in the length of the measured groups from the background of human protein coding genes [60], relative to the observed overlap, repeated 10,000 times. Additional statistical analysis was performed using GraphPad Prism version 10.0.0, Boston, Massachusetts, USA, www.graphpad.com, and included either t-test, one-way ANOVA or two-way ANOVA followed by post hoc Dunnet, Sidak, or Tukey test. Statistical significance was determined when *P*-value was below 0.05 (detailed statistical analysis per experiment in Table S6).

### STAR★Methods

Key resources table are in Supplementary Table 9.

Table S6 contains all the statistical tests done per figure.

## Supplementary information

supplmemental figures

Suplementary material and resource table

S1

S2

S3

S4

S5

S6

S7

S8

Material and Methods

Original Western blot images

## Data Availability

ATAC-seq and ChIP-seq data have been deposited at GEO and are publicly available as of the date of publication. Accession numbers are listed in the key resources table. Any additional information required to reanalyze the data reported in this work paper is available from the lead contact upon request.

## Materials availability

Plasmids generated in this study are available and can be requested from the lead author with a completed Material Transfer Agreement.

## Code availability

Original code was deposited at Zenodo and is publicly available as of the data of publication. DOIs are listed in the key resources table.

## Resource availability

The lead contact, Debra Toiber (toiber@bgu.ac.il), should be contacted for requests regarding resources, data, and reagents used in the study.
