# Supplementary material for: SIRT6-dependent functional switch via K494 modifications of RE-1 silencing transcription factor
Source: Cell Death Dis. 2024 Nov 7;15(11):798. doi: 10.1038/s41419-024-07160-0 (PMC11543946; doi:10.1038/s41419-024-07160-0)
Supplement: Supplementary file 1 — supplmemental figures [file 41419_2024_7160_MOESM1_ESM.pdf]

Supplementary Figure 1

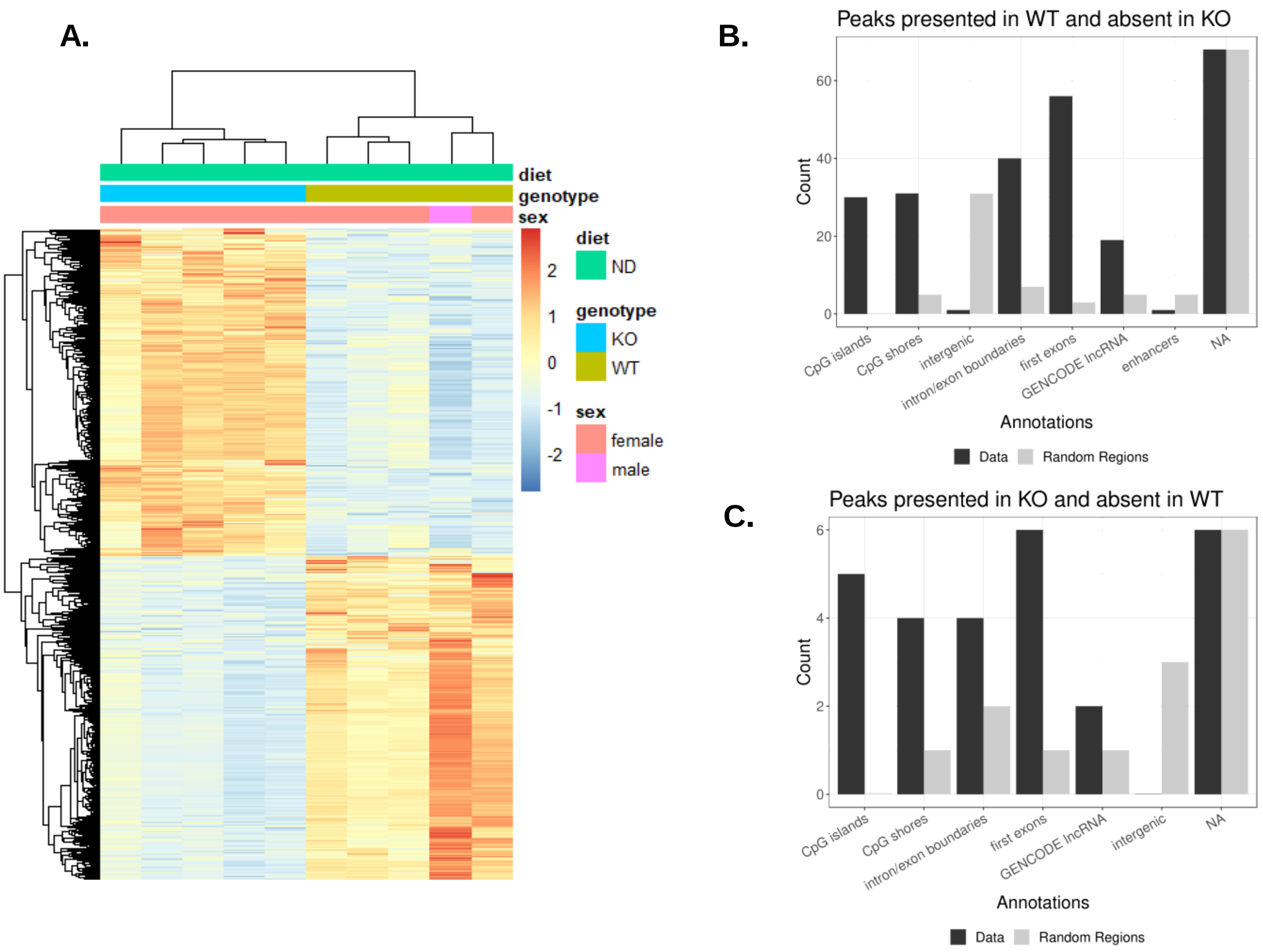

Figure S1 Neural genes are upregulated in brS6KO brain. **(A)** Heatmap of the differentially expressed genes between WT and KO (brS6KO) mice brains RNA-seq. **(B)** Normalized counts of annotated peaks in WT cortical neurons. **(C)** Normalized counts of annotated peaks in KO cortical neurons.

Supplementary Figure 2

A.

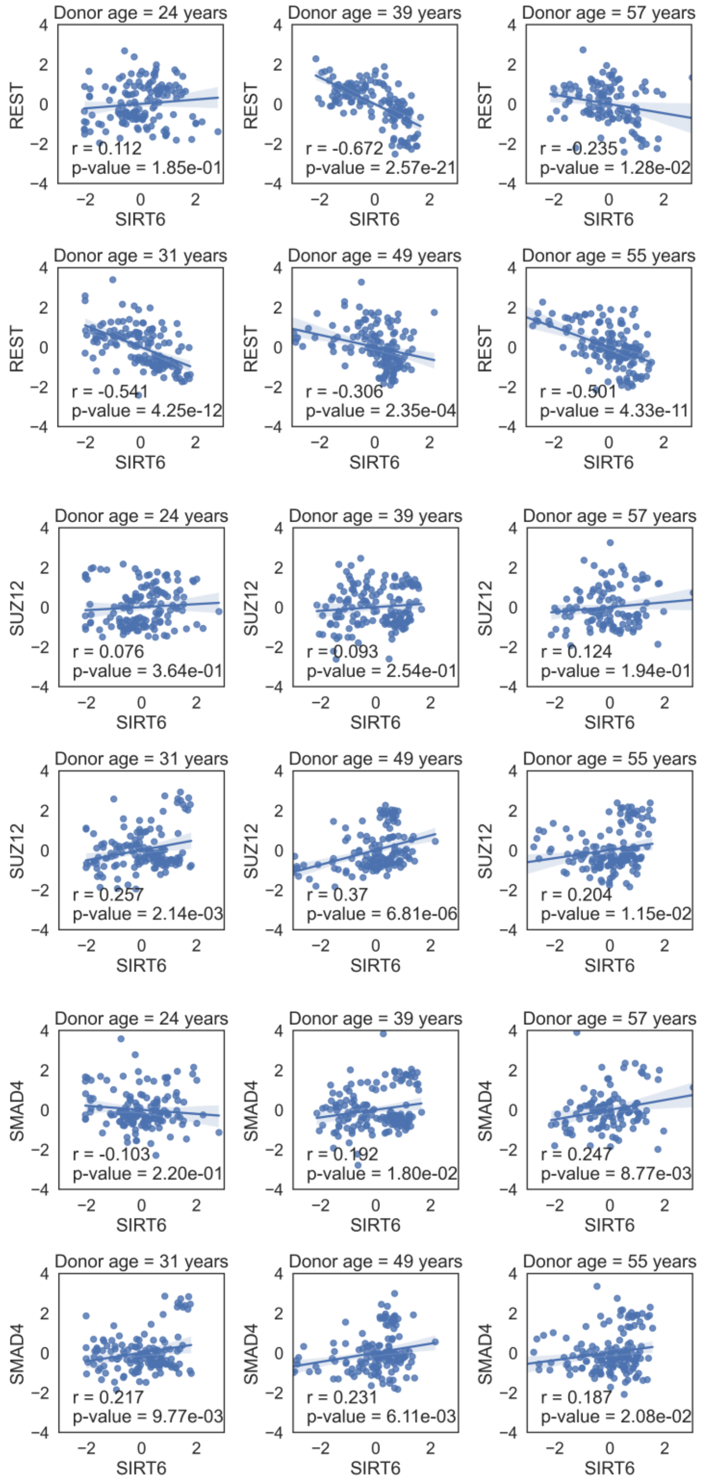

Figure S2: REST regulates upregulated genes in SIRT6 KO. **(A)** Expression profile correlation between SIRT6 and each of the candidate transcription factors in six human brains, each individual brain is visualized. Each dot represents the expression profile of SIRT6 and the TF in question in a particular brain region. r = Pearson R.

Supplementary Figure 3

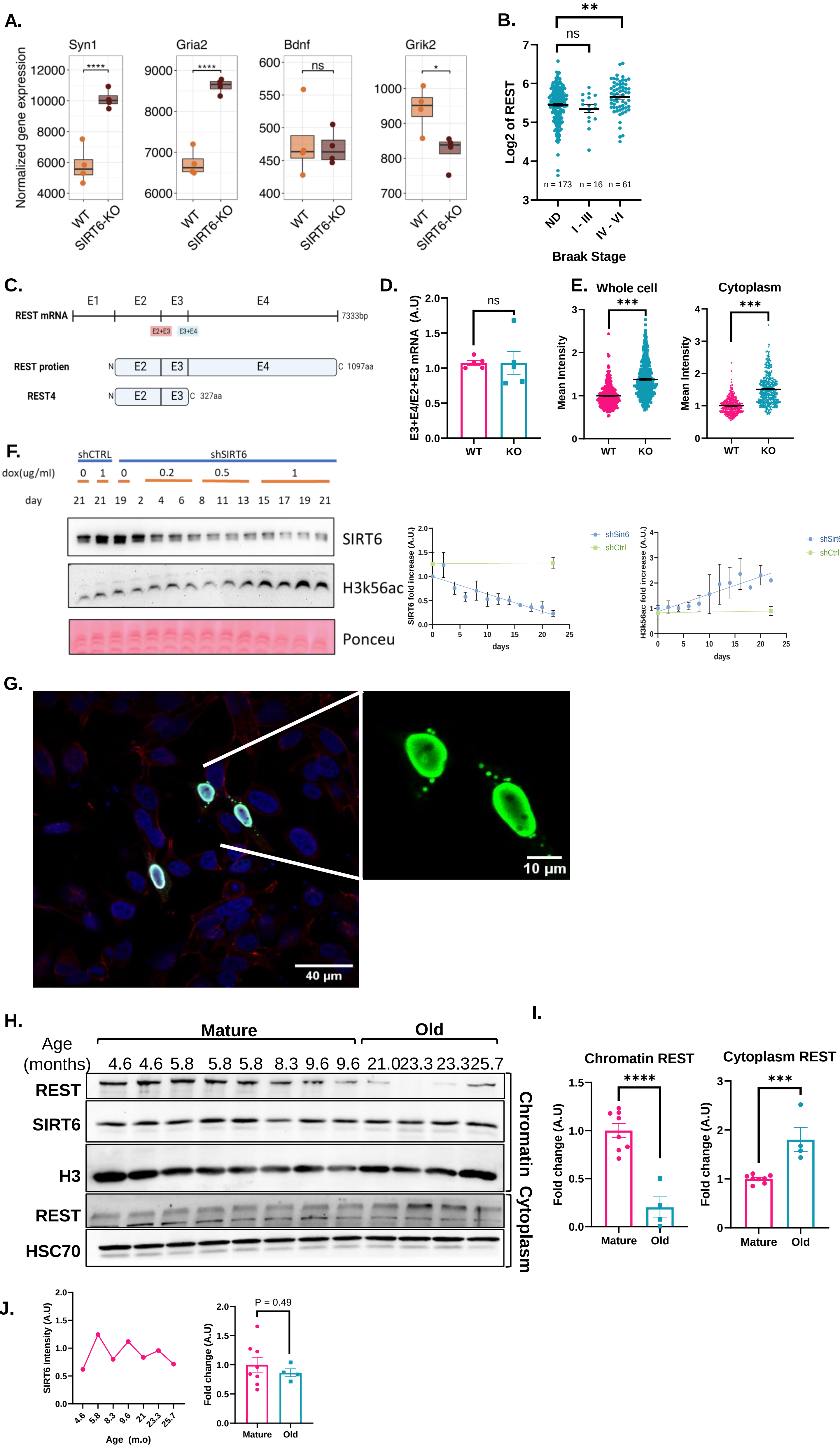

Figure S3: REST expression is upregulated in SIRT6 KO.

(A) REST target genes normalized expression from mouse brain RNA-seq. n(mice)=5. **(B)** REST expression in Alzheimer patient brains, relative to Braak stage. n(patients). **(C-D)** Relative REST isoform levels in WT vs KO SHSY-5 cells. E3+E4 represent primers targeting REST main isoform. E2+E3 represents primers targeting a shared region between the main isoform and REST4 isoform. n(replicates)=5. **(E)** Quantification of whole cell and cytoplasmic REST intensity in WT and KO SHSY-5Y cells. n(cells)=326[WT]337[KO]. **(F)** WB of SIRT6 and H3K56ac in inducible shSIRT6 SHSY-5Y cells chromatin fraction. Days represent the day on induction. Dox represents the concentration of doxycycline treatment in µg/ml. Quantification of SIRT6 and H3K56ac according to F. **(G)** IF of SHSY-5Y cells transfected with Flag-REST-GFP construct. **(H-J)** Chromatin and cytoplasmic of mature and old mice left cortexes. J represents SIRT6 quantification according to the WB. n(mice)=8(Mature),4(Old). Data is represented as Mean±SEM. D, I-J Unpaired t-test. B One-way ANOVA followed by Dunnett's multiple comparisons test. Two tailed *p<0.05, **p<0.01, ***p<0.001, ****p<0.0001. ns=non significant.

Supplementary Figure 4

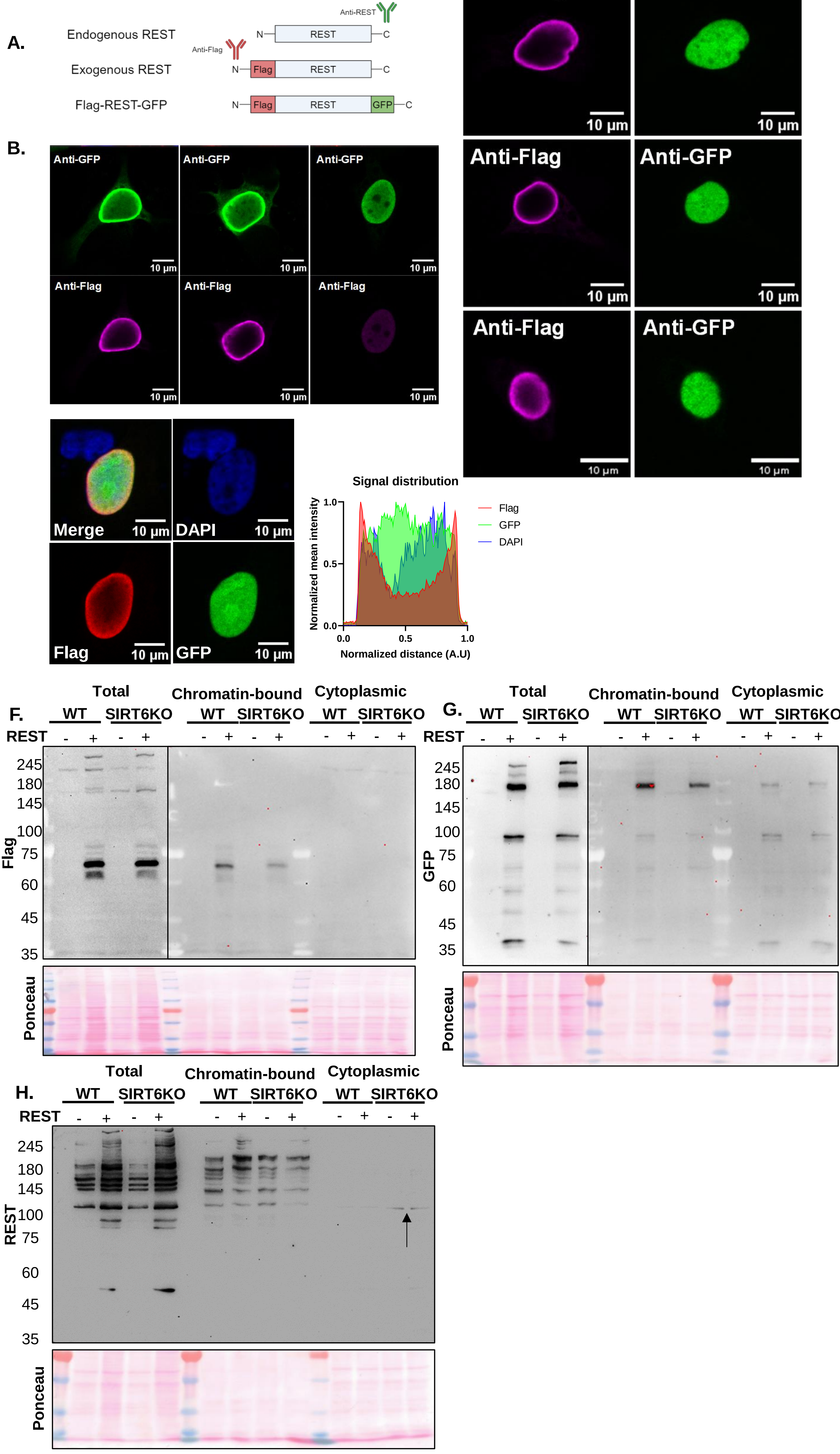

Figure S4: REST localization alters in SIRT6 KO.

(A) Illustration of the differences in recognition of endogenous REST protein, Flag-REST exogenous protein and the Flag-REST-GFP model. (B) SHSY-5Y cell transfected with Flag-REST-GFP. Flag channel in red, GFP in green, DAPI in blue. Signal distribution plot of the nucleus cross section is represented on the right. (F-H) WB of the total, chromatin-bound or cytoplasmic protein fractions extracted from WT and SIRT6 KO SHSY-5Y cells transfected with empty vector (EV) or with Flag-REST-GFP. The top membrane is anti-Flag (I) or anti-GFP (J) or anti-REST (H). The bottom membrane is ponceau staining. Arrows point on two bands representing REST in the cytoplasmic fraction of SIRT6 KO samples. The numbers on the left side represent the kDa ladder.

Supplementary Figure 5

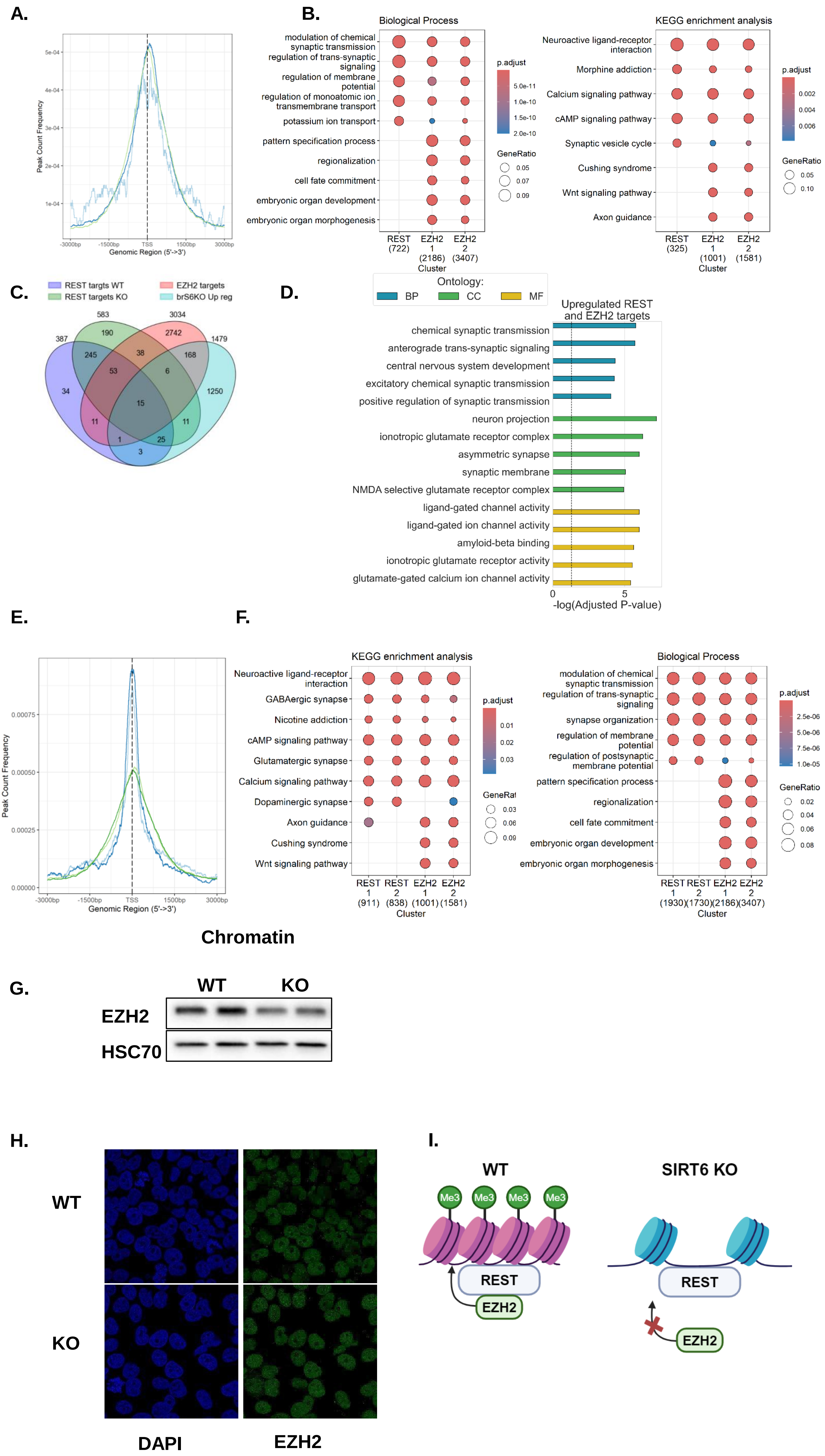

Figure S5: REST loses interaction with EZH2 in SIRT6 KO.

(A) Peak frequency around transcription start site (TSS) of consensus REST peaks and 2 EZH2 replicates from hESC. (B) Biological processes and KEGG pathways enriched for REST and EZH2 peaks. (C) Venn diagram representing cross between REST consensus peaks in WT and KO cells, brS6KO upregulated genes and EZH2 targets in hESC. Biological processes enriched for REST and EZH2 peaks. (D) Enrichment analysis of brS6KO upregulated genes targeted by REST and EZH2 in Fig. 5D. (E) Peak frequency of REST and EZH2 in two replicates of hESC ChIP-seq. **(F)** Functional enrichment analysis of REST and EZH2 targets in GO biological processes and KEGG pathways. (G) Chromatin extraction of SHSY-5Y cells. EZH2 levels are lower in SIRT6 KO chromatin compared to WT. **(H)** Immunofluorescence of EZH2 in WT and SIRT6 KO KO SHSY-Y cells. EZH2 channel in green, DAPI channel in blue. (I) Schematic representation of REST binding to chromatin in WT and SIRT6 KO background, interacting with EZH2 and H3K27me3. BP = Biological Processes, CC = Cellular Component, MF = Molecular Function. The dashed line represents Adjusted P-value=0.05.

Supplementary Figure 6

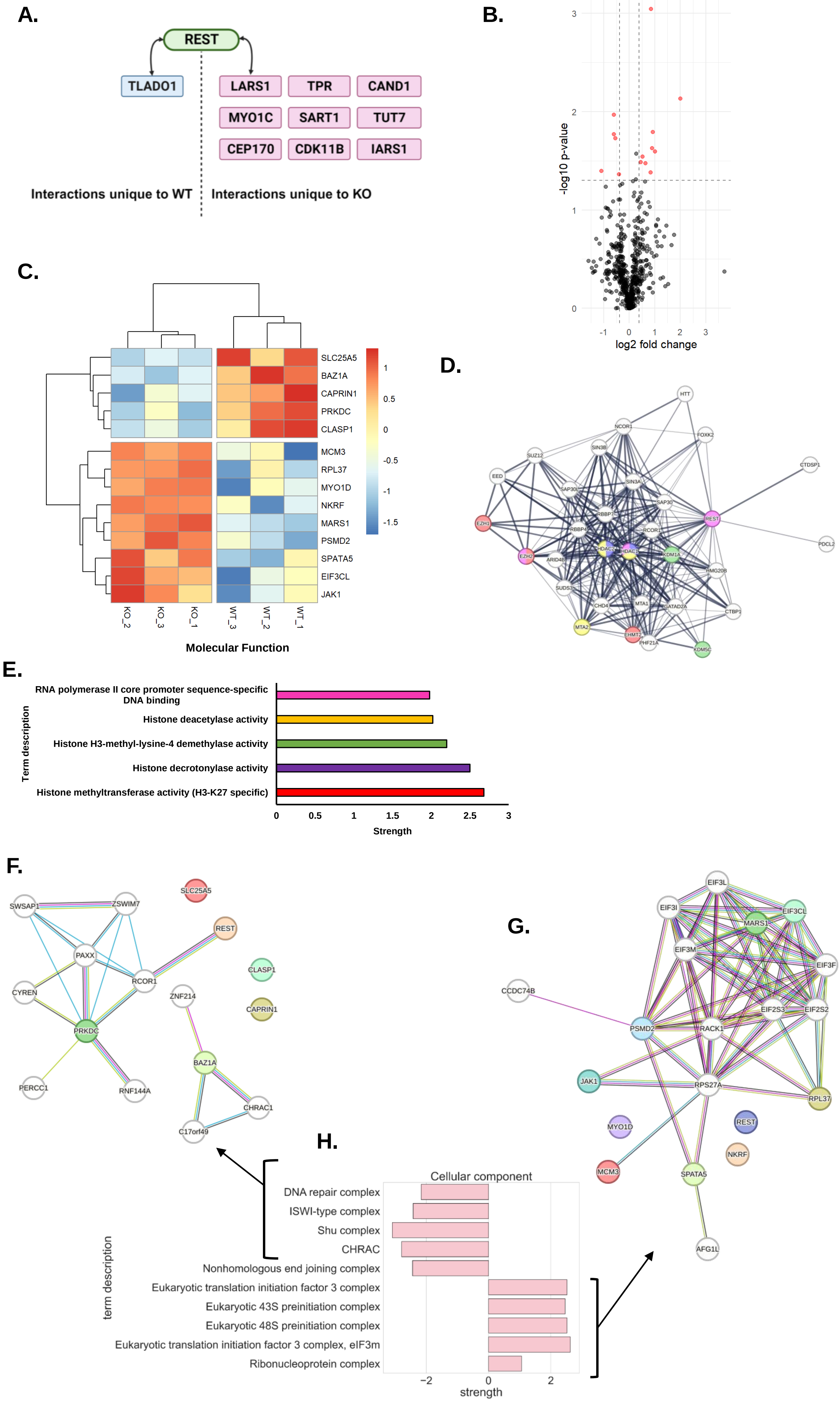

Figure S6: REST differential interaction in WT and SIRT6KO HEK293T cell line.

(A) Unique interactions with REST in SIRT6 WT and KO cells. **(B)** Volcano plot of differential interactions with REST immunoprecipitated form HEK293T cells. **(C)** Cluster heatmap proteins interacting with REST in WT and SIRT6 KO cells. **(D-E)** String analysis of physical REST interactors. Proteins in D are colored according to the enriched categories they represent in E. (F-G) STRING analysis of REST lost (F) and gained (G) interactions in SIRT6 KO cells. Bar plots represent cellular component categories lost or gained. (H) Cellular component enrichment analysis for lost and gained interaction networks.

Supplementary Figure 7

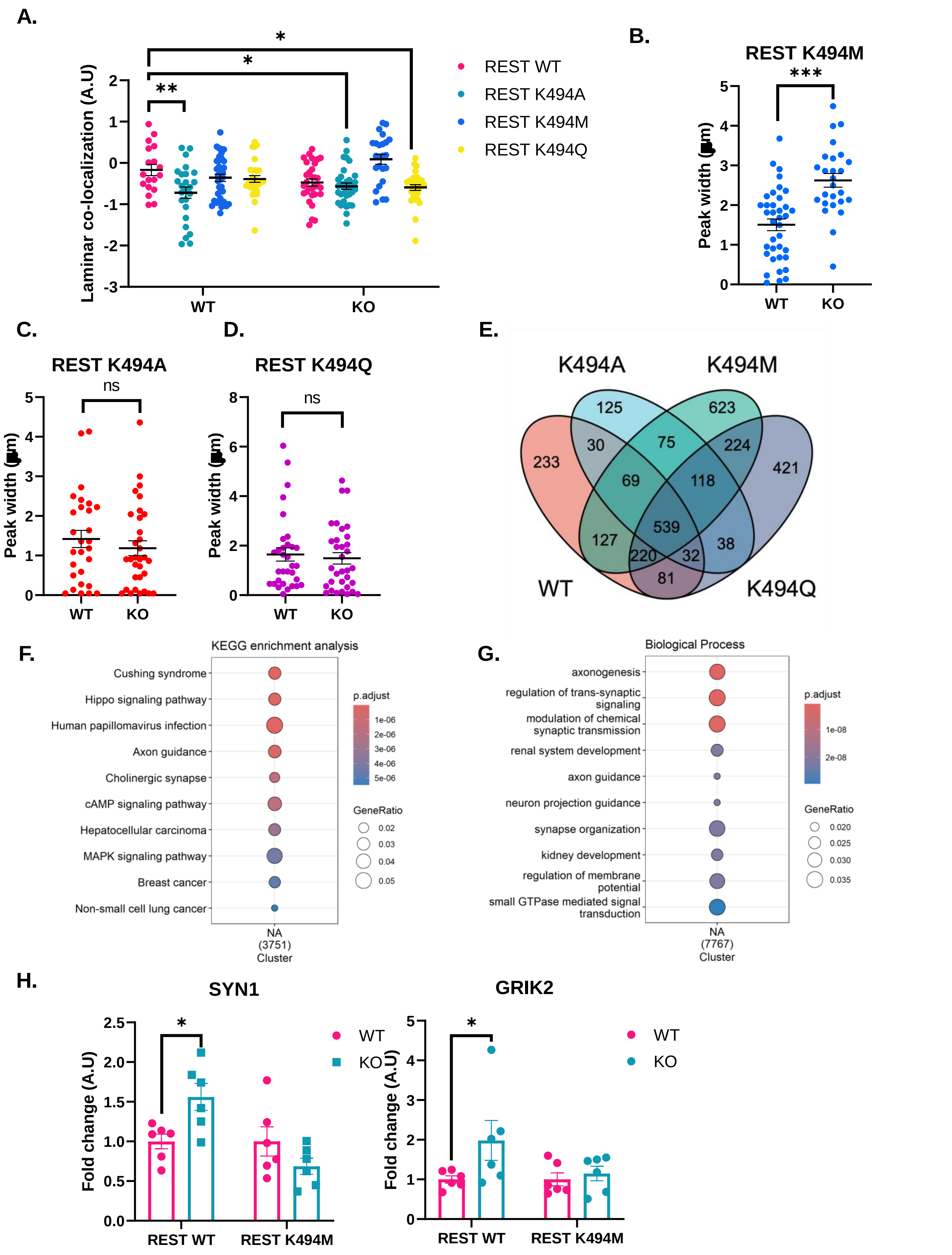

Figure S7: Peak width in REST mutants. (A) Quantification of REST nuclear Laminar co-localization value relative to LAMINB and DAPI staining. n(cells)= 18[WT:REST WT], 25[WT:REST K494A], 36[WT:K494M], 30[WT:K494Q 31[KO:REST WT], 33[KO:REST K494A], 24[WT:K494M], 31[KO:REST K494Q]. (B) Peak width of REST K494M methyl mimic mutant. n(cells)= WT[31],KO[26]. (C-D) Peak width of REST K494A and K494Q mutants in SHSY-5Y cells (E) Venn diagram for 2 out of 3 replicates for each REST mutant. (F-G) Functional enrichment analysis for pathways (F) and Biological processes (G) enriched in REST WT, K494A, K494M and K494Q ChIP-seq consensus peaks. (H) mRNA levels of SYN1 and GRIK2 in SHSY-5Y cells transfected with exogenous REST, WT or K494M mutants. n(replicates)=6. Data represented as Mean±SEM. A-E 2-Way ANOVA followed by Dunnett’s or Sidak’s multiple comparison test. *p<0.05, **p<0.01, ***p<0.001.
