# Supplementary material for: SIRT6-dependent functional switch via K494 modifications of RE-1 silencing transcription factor
Source: Cell Death Dis. 2024 Nov 7;15(11):798. doi: 10.1038/s41419-024-07160-0 (PMC11543946; doi:10.1038/s41419-024-07160-0)
Supplement: Supplementary file 2 — Suplementary material and resource table [file 41419_2024_7160_MOESM2_ESM.docx]

# Key resources table

| REAGENT or RESOURCE | SOURCE | IDENTIFIER |
| --- | --- | --- |
| Antibodies | | |
| REST antibody | Proteintech | Cat# 22242-1-AP, RRID: AB_2879044 |
| Rabbit Anti-Human REST / NRSF Polyclonal Antibody, Unconjugated | Abcam | Cat# ab21635, RRID:AB_777678 |
| Rabbit Anti-REST ChIPAb+ Polyclonal Antibody, Unconjugated | Millipore | Cat# 17-641, RRID:AB_1977463 |
| Anti-Lamin B1 antibody - Nuclear Envelope Marker | Abcam | Cat# ab16048, RRID:AB_443298 |
| β-Actin (13E5) Rabbit mAb | Cell Signaling Technology | Cat# 4970 (also 4970P, 4970L, 4970S), RRID:AB_2223172 |
| Anti-Histone H3 antibody - Nuclear Loading Control and ChIP Grade | Abcam | Cat# ab1791, RRID:AB_302613 |
| Fibrillarin antibody [38F3] - Nucleolar Marker | Abcam | Cat# ab4566, RRID:AB_304523 |
| EZH2-human | Cell Signaling Technology | Cat# 5246 (also ENCAB350TQZ, 5246BF, 5246S, 5246P), RRID:AB_10694683 |
| Monoclonal ANTI-FLAG® M2 antibody produced in mouse | Sigma-Aldrich | Cat# F1804, RRID:AB_262044 |
| Rabbit Anti-Methylated Lysine (mono methyl, di methyl) Polyclonal Antibody, Unconjugated | Abcam | Cat# ab23366, RRID:AB_447401 |
| Rabbit Anti-Lysine, acetyl Polyclonal Antibody, Unconjugated | Abcam | Cat# ab21623, RRID:AB_446436 |
| Rabbit Anti-Histone H3, Trimethyl (Lys27) Monoclonal Antibody, Unconjugated, Clone C36B11 | Cell Signaling Technology | Cat# 9733 (also 9733S, 9733P), RRID:AB_2616029 |
| Rabbit Anti-Hsc70 Polyclonal Antibody, Unconjugated | Abcam | Cat# ab1427, RRID:AB_301048 |
| Anti-GFP | Roche | Cat# 11814460001, RRID:AB_390913 |
| Anti-LC3B | Sigma-Aldrich | Cat# L7543, RRID:AB_796155 |
| Alexa Fluor 647-AffiniPure Donkey Anti-Rabbit IgG (H+L) | Jackson ImmunoResearch Labs | Cat# 711-605-152, RRID:AB_2492288 |
| Alexa Fluor® 594 AffiniPure Donkey Anti-Rabbit IgG (H+L) | Jackson ImmunoResearch Labs | Cat# 711-585-152, RRID:AB_2340621 |
| Donkey Anti-Mouse IgG H&L (Alexa Fluor® 555) preadsorbed | Abcam | Cat# ab150110, RRID:AB_2783637 |
| Alexa Fluor 488 AffiniPure Donkey Anti-Rabbit IgG (H+L) | Jackson ImmunoResearch Labs | Cat# 711-545-152, RRID:AB_2313584 |
| Rabbit anti-Mouse IgG H&L (HRP) secondary antibody | Abcam | Cat# ab97046, RRID:AB_10680920 |
| Goat Anti-Rabbit IgG H&L (HRP) | Abcam | Cat# ab6721, RRID:AB_955447 |
| Critical commercial assays | | |
| EZ-RNA II | Biological Industries | Cat# 20-410-100 |
| NucleoSpin® RNA kit | MACEHERY-NAGEL | Cat# 740984.50 |
| Nucleo Spin Gel and PCR Clean-Up kit | MACEHERY-NAGEL | Cat# 740609 |
| Anti-FLAG® M2 Magnetic Beads | Millipore ® | Cat# M8823 |
| SureBeads™ Protein G Magnetic Beads | BIO-RAD | Cat# 1614023 |
| SureBeads™ Protein A Magnetic Beads | BIO-RAD | Cat# 1614013 |
| RNeasy MinElute Cleanup Kit | QIAGEN | Cat# 74204 |
| qScript cDNA Synthesis Kit | QuantaBio | Cat# 95047-100 |
| ROCHE LightCycler® 480Probes Master | Roche | Cat# 04902343001 |
| BIO-RAD SsoAdvanced Universal SYBR® Green Supermix | BIO-RAD | Cat# 1725275 |
| PolyJet™ In Vitro DNA Transfection Reagent | SignaGen Laboratories | Cat# SL100688 |
| Deposited data | | |
| ATAC-seq of cortical neurons | This study |  |
| ChIP-seq of endogenous REST in WT and SIRT6KO SHSY-5Y cells | This study |  |
| ChIP-seq of REST WT, K494A, K494M, K494Q in SHSY-5Y cells | This study |  |
| REST Mass spectrometry in WT and SIRT6 KO H293T cells | This study |  |
| Experimental models: Cell lines | | |
| CRISPR control and SIRT6 KO SHSY-5Y cell line | Toiber lab | N/A |
| CRISPR control and SIRT6 KO HEK293T cell line | Toiber lab | N/A |
| shSIRT6 and shCtrl SHSY-5Y cell line | Mostoslavsky lab | N/A |
| Experimental models: Organisms/strains | | |
| WT C57BL6 mice | Jackson Laboratories | RRID:IMSR_JAX:000664 |
| WT (cre-) and brS6KO | Toiber lab |  |
| Oligonucleotides | | |
| RT-qPCR Primers | Table S7 |  |
| REST mutagenesis primers | Table S8 |  |
| Recombinant DNA | | |
| LPC-flag-REST-WT | Addgene | RRID:Addgene_41903 |
| pET28 hSIRT6-His | Haim Cohen lab | N/A |
| CMV-Flag | Mostoslavsky lab | N/A |
| LPC-flag-REST-K494A | Toiber lab | N/A |
| LPC-flag-REST-K494M | Toiber lab | N/A |
| LPC-flag-REST-K494Q | Toiber lab | N/A |
| LPC-flag-REST-GFP | Toiber lab | N/A |
| Software and algorithms | | |
| ImageJ (FIJI) | Schindelin et al.(30) | https://fiji.sc/ |
| GraphPad prism version 10.0.0 | Software | https://www.graphpad.com/features |
| Cell profiler | Stirling et al.(31) | https://cellprofiler.org/releases |
| Spyder | Software | https://www.spyder-ide.org/ |
| R studio | Software | https://posit.co/downloads/ |
| Original code | DOI(32) |  |

| REAGENT or RESOURCE | SOURCE | IDENTIFIER |
| --- | --- | --- |
| Antibodies | | |
| REST antibody | Proteintech | Cat# 22242-1-AP, RRID: AB_2879044 |
| Rabbit Anti-Human REST / NRSF Polyclonal Antibody, Unconjugated | Abcam | Cat# ab21635, RRID:AB_777678 |
| Rabbit Anti-REST ChIPAb+ Polyclonal Antibody, Unconjugated | Millipore | Cat# 17-641, RRID:AB_1977463 |
| Anti-Lamin B1 antibody - Nuclear Envelope Marker | Abcam | Cat# ab16048, RRID:AB_443298 |
| β-Actin (13E5) Rabbit mAb | Cell Signaling Technology | Cat# 4970 (also 4970P, 4970L, 4970S), RRID:AB_2223172 |
| Anti-Histone H3 antibody - Nuclear Loading Control and ChIP Grade | Abcam | Cat# ab1791, RRID:AB_302613 |
| Fibrillarin antibody [38F3] - Nucleolar Marker | Abcam | Cat# ab4566, RRID:AB_304523 |
| EZH2-human | Cell Signaling Technology | Cat# 5246 (also ENCAB350TQZ, 5246BF, 5246S, 5246P), RRID:AB_10694683 |
| Monoclonal ANTI-FLAG® M2 antibody produced in mouse | Sigma-Aldrich | Cat# F1804, RRID:AB_262044 |
| Rabbit Anti-Methylated Lysine (mono methyl, di methyl) Polyclonal Antibody, Unconjugated | Abcam | Cat# ab23366, RRID:AB_447401 |
| Rabbit Anti-Lysine, acetyl Polyclonal Antibody, Unconjugated | Abcam | Cat# ab21623, RRID:AB_446436 |
| Rabbit Anti-Histone H3, Trimethyl (Lys27) Monoclonal Antibody, Unconjugated, Clone C36B11 | Cell Signaling Technology | Cat# 9733 (also 9733S, 9733P), RRID:AB_2616029 |
| Rabbit Anti-Hsc70 Polyclonal Antibody, Unconjugated | Abcam | Cat# ab1427, RRID:AB_301048 |
| Anti-GFP | Roche | Cat# 11814460001, RRID:AB_390913 |
| Anti-LC3B | Sigma-Aldrich | Cat# L7543, RRID:AB_796155 |
| Alexa Fluor 647-AffiniPure Donkey Anti-Rabbit IgG (H+L) | Jackson ImmunoResearch Labs | Cat# 711-605-152, RRID:AB_2492288 |
| Alexa Fluor® 594 AffiniPure Donkey Anti-Rabbit IgG (H+L) | Jackson ImmunoResearch Labs | Cat# 711-585-152, RRID:AB_2340621 |
| Donkey Anti-Mouse IgG H&L (Alexa Fluor® 555) preadsorbed | Abcam | Cat# ab150110, RRID:AB_2783637 |
| Alexa Fluor 488 AffiniPure Donkey Anti-Rabbit IgG (H+L) | Jackson ImmunoResearch Labs | Cat# 711-545-152, RRID:AB_2313584 |
| Rabbit anti-Mouse IgG H&L (HRP) secondary antibody | Abcam | Cat# ab97046, RRID:AB_10680920 |
| Goat Anti-Rabbit IgG H&L (HRP) | Abcam | Cat# ab6721, RRID:AB_955447 |
| Critical commercial assays | | |
| EZ-RNA II | Biological Industries | Cat# 20-410-100 |
| NucleoSpin® RNA kit | MACEHERY-NAGEL | Cat# 740984.50 |
| Nucleo Spin Gel and PCR Clean-Up kit | MACEHERY-NAGEL | Cat# 740609 |
| Anti-FLAG® M2 Magnetic Beads | Millipore ® | Cat# M8823 |
| SureBeads™ Protein G Magnetic Beads | BIO-RAD | Cat# 1614023 |
| SureBeads™ Protein A Magnetic Beads | BIO-RAD | Cat# 1614013 |
| RNeasy MinElute Cleanup Kit | QIAGEN | Cat# 74204 |
| qScript cDNA Synthesis Kit | QuantaBio | Cat# 95047-100 |
| ROCHE LightCycler® 480Probes Master | Roche | Cat# 04902343001 |
| BIO-RAD SsoAdvanced Universal SYBR® Green Supermix | BIO-RAD | Cat# 1725275 |
| PolyJet™ In Vitro DNA Transfection Reagent | SignaGen Laboratories | Cat# SL100688 |
| Deposited data | | |
| ATAC-seq of cortical neurons | This study |  |
| ChIP-seq of endogenous REST in WT and SIRT6KO SHSY-5Y cells | This study |  |
| ChIP-seq of REST WT, K494A, K494M, K494Q in SHSY-5Y cells | This study |  |
| REST Mass spectrometry in WT and SIRT6 KO H293T cells | This study |  |
| Experimental models: Cell lines | | |
| CRISPR control and SIRT6 KO SHSY-5Y cell line | Toiber lab | N/A |
| CRISPR control and SIRT6 KO HEK293T cell line | Toiber lab | N/A |
| shSIRT6 and shCtrl SHSY-5Y cell line | Mostoslavsky lab | N/A |
| Experimental models: Organisms/strains | | |
| WT C57BL6 mice | Jackson Laboratories | RRID:IMSR_JAX:000664 |
| WT (cre-) and brS6KO | Toiber lab |  |
| Oligonucleotides | | |
| RT-qPCR Primers | Table S7 |  |
| REST mutagenesis primers | Table S8 |  |
| Recombinant DNA | | |
| LPC-flag-REST-WT | Addgene | RRID:Addgene_41903 |
| pET28 hSIRT6-His | Haim Cohen lab | N/A |
| CMV-Flag | Mostoslavsky lab | N/A |
| LPC-flag-REST-K494A | Toiber lab | N/A |
| LPC-flag-REST-K494M | Toiber lab | N/A |
| LPC-flag-REST-K494Q | Toiber lab | N/A |
| LPC-flag-REST-GFP | Toiber lab | N/A |
| Software and algorithms | | |
| ImageJ (FIJI) | Schindelin et al.(30) | https://fiji.sc/ |
| GraphPad prism version 10.0.0 | Software | https://www.graphpad.com/features |
| Cell profiler | Stirling et al.(31) | https://cellprofiler.org/releases |
| Spyder | Software | https://www.spyder-ide.org/ |
| R studio | Software | https://posit.co/downloads/ |
| Original code | DOI(32) |  |
