# Supplementary material for: SIRT6-dependent functional switch via K494 modifications of RE-1 silencing transcription factor
Source: Cell Death Dis. 2024 Nov 7;15(11):798. doi: 10.1038/s41419-024-07160-0 (PMC11543946; doi:10.1038/s41419-024-07160-0)
Supplement: Supplementary file 11 — Material and Methods [file 41419_2024_7160_MOESM11_ESM.docx]

STAR★Methods

# Key resources table

| REAGENT or RESOURCE | SOURCE | IDENTIFIER |
| --- | --- | --- |
| Antibodies | | |
| REST antibody | Proteintech | Cat# 22242-1-AP, RRID: AB_2879044 |
| Rabbit Anti-Human REST / NRSF Polyclonal Antibody, Unconjugated | Abcam | Cat# ab21635, RRID:AB_777678 |
| Rabbit Anti-REST ChIPAb+ Polyclonal Antibody, Unconjugated | Millipore | Cat# 17-641, RRID:AB_1977463 |
| Anti-Lamin B1 antibody - Nuclear Envelope Marker | Abcam | Cat# ab16048, RRID:AB_443298 |
| β-Actin (13E5) Rabbit mAb | Cell Signaling Technology | Cat# 4970 (also 4970P, 4970L, 4970S), RRID:AB_2223172 |
| Anti-Histone H3 antibody - Nuclear Loading Control and ChIP Grade | Abcam | Cat# ab1791, RRID:AB_302613 |
| Fibrillarin antibody [38F3] - Nucleolar Marker | Abcam | Cat# ab4566, RRID:AB_304523 |
| EZH2-human | Cell Signaling Technology | Cat# 5246 (also ENCAB350TQZ, 5246BF, 5246S, 5246P), RRID:AB_10694683 |
| Monoclonal ANTI-FLAG® M2 antibody produced in mouse | Sigma-Aldrich | Cat# F1804, RRID:AB_262044 |
| Rabbit Anti-Methylated Lysine (mono methyl, di methyl) Polyclonal Antibody, Unconjugated | Abcam | Cat# ab23366, RRID:AB_447401 |
| Rabbit Anti-Lysine, acetyl Polyclonal Antibody, Unconjugated | Abcam | Cat# ab21623, RRID:AB_446436 |
| Rabbit Anti-Histone H3, Trimethyl (Lys27) Monoclonal Antibody, Unconjugated, Clone C36B11 | Cell Signaling Technology | Cat# 9733 (also 9733S, 9733P), RRID:AB_2616029 |
| Rabbit Anti-Hsc70 Polyclonal Antibody, Unconjugated | Abcam | Cat# ab1427, RRID:AB_301048 |
| Anti-GFP | Roche | Cat# 11814460001, RRID:AB_390913 |
| Anti-LC3B | Sigma-Aldrich | Cat# L7543, RRID:AB_796155 |
| Alexa Fluor 647-AffiniPure Donkey Anti-Rabbit IgG (H+L) | Jackson ImmunoResearch Labs | Cat# 711-605-152, RRID:AB_2492288 |
| Alexa Fluor® 594 AffiniPure Donkey Anti-Rabbit IgG (H+L) | Jackson ImmunoResearch Labs | Cat# 711-585-152, RRID:AB_2340621 |
| Donkey Anti-Mouse IgG H&L (Alexa Fluor® 555) preadsorbed | Abcam | Cat# ab150110, RRID:AB_2783637 |
| Alexa Fluor 488 AffiniPure Donkey Anti-Rabbit IgG (H+L) | Jackson ImmunoResearch Labs | Cat# 711-545-152, RRID:AB_2313584 |
| Rabbit anti-Mouse IgG H&L (HRP) secondary antibody | Abcam | Cat# ab97046, RRID:AB_10680920 |
| Goat Anti-Rabbit IgG H&L (HRP) | Abcam | Cat# ab6721, RRID:AB_955447 |
| Critical commercial assays | | |
| EZ-RNA II | Biological Industries | Cat# 20-410-100 |
| NucleoSpin® RNA kit | MACEHERY-NAGEL | Cat# 740984.50 |
| Nucleo Spin Gel and PCR Clean-Up kit | MACEHERY-NAGEL | Cat# 740609 |
| Anti-FLAG® M2 Magnetic Beads | Millipore ® | Cat# M8823 |
| SureBeads™ Protein G Magnetic Beads | BIO-RAD | Cat# 1614023 |
| SureBeads™ Protein A Magnetic Beads | BIO-RAD | Cat# 1614013 |
| RNeasy MinElute Cleanup Kit | QIAGEN | Cat# 74204 |
| qScript cDNA Synthesis Kit | QuantaBio | Cat# 95047-100 |
| ROCHE LightCycler® 480Probes Master | Roche | Cat# 04902343001 |
| BIO-RAD SsoAdvanced Universal SYBR® Green Supermix | BIO-RAD | Cat# 1725275 |
| PolyJet™ In Vitro DNA Transfection Reagent | SignaGen Laboratories | Cat# SL100688 |
| Deposited data | | |
| ATAC-seq of cortical neurons | This study |  |
| ChIP-seq of endogenous REST in WT and SIRT6KO SHSY-5Y cells | This study |  |
| ChIP-seq of REST WT, K494A, K494M, K494Q in SHSY-5Y cells | This study |  |
| REST Mass spectrometry in WT and SIRT6 KO H293T cells | This study |  |
| Experimental models: Cell lines | | |
| CRISPR control and SIRT6 KO SHSY-5Y cell line | Toiber lab | N/A |
| CRISPR control and SIRT6 KO HEK293T cell line | Toiber lab | N/A |
| shSIRT6 and shCtrl SHSY-5Y cell line | Mostoslavsky lab | N/A |
| Experimental models: Organisms/strains | | |
| WT C57BL6 mice | Jackson Laboratories | RRID:IMSR_JAX:000664 |
| WT (cre-) and brS6KO | Toiber lab |  |
| Oligonucleotides | | |
| RT-qPCR Primers | Table S7 |  |
| REST mutagenesis primers | Table S8 |  |
| Recombinant DNA | | |
| LPC-flag-REST-WT | Addgene | RRID:Addgene_41903 |
| pET28 hSIRT6-His | Haim Cohen lab | N/A |
| CMV-Flag | Mostoslavsky lab | N/A |
| LPC-flag-REST-K494A | Toiber lab | N/A |
| LPC-flag-REST-K494M | Toiber lab | N/A |
| LPC-flag-REST-K494Q | Toiber lab | N/A |
| LPC-flag-REST-GFP | Toiber lab | N/A |
| Software and algorithms | | |
| ImageJ (FIJI) | Schindelin et al.^28^ | https://fiji.sc/ |
| GraphPad prism version 10.0.0 | Software | https://www.graphpad.com/features |
| Cell profiler | Stirling et al.^29^ | https://cellprofiler.org/releases |
| Spyder | Software | https://www.spyder-ide.org/ |
| R studio | Software | https://posit.co/downloads/ |
| Original code | DOI^30^ |  |

Resource availability

Lead contact.

The lead contact, Debra Toiber (toiber@bgu.ac.il), should be contacted for requests regarding resources, data, and reagents used in the study.

Materials availability

Plasmids generated in this study are available and can be requested from the lead author with a completed Material Transfer Agreement.

Data and code availability

- ATAC-seq and ChIP-seq data have been deposited at GEO and are publicly available as of the date of publication. Accession numbers are listed in the key resources table.
- Original code was deposited at Zenodo and is publicly available as of the data of publication. DOIs are listed in the key resources table.
- Any additional information required to reanalyze the data reported in this work paper is available from the lead contact upon request.

Experimental model and study participant details

Mice

Mice models were WT C57BL6 mice (Jackson laboratories) and WT (Cre-) and brSIRT6 KO mice developed in Dr. Toibers Lab.

Cell lines

Cell lines used in the study are described in the Key Resources table. All cell lines were cultured and maintained in 37° C, 5% CO_2_ incubation. Dulbecco's Modified Eagle Medium (DMEM) (#Cat: 11965092, Gibco^TM^) supplemented with 5% L-glutamine (#Cat: A2916801, Gibco^TM^), 5% Penicillin-Streptomycin (#Cat: 15140122, Gibco^TM^) and 10% heat inactivated Fetal Bovine Serum (#Cat: 10082147, Gibco^TM^).

Generation of brS6KO Mice

SIRT6 KO mice used of this study were generated previously by Kaluski et al.^31^ In brief, Sirt6 conditional vector was inserted in a Neo cassette (flanked by two Frt seq) together with Sirt6 exon2 flanked by two loxP sites. Targeted ES cells (V6.5) were injected into C57BL6/J blastocysts. The Neo cassette was deleted in vivo by crossing the chimeras with a mouse expressing the Flpe endonuclease. Mice were backcrossed for 3 generations with C57BL6/J mice to obtain heterozygous mice that were 97% C57BL6/J background. These mice were bred with C57BL/Nestin-Cre/J mice (Jackson Laboratories).

RNA-seq data analysis

The RNA-seq data was used from a previously published work (GSE22107)^32^. For this analysis, mice only under normal diet (ND) were analyzed. Analysis was done based on the aligned reads of 5 WT and 5 brS6KO mice. RNA-seq differential expression (DE) analysis was performed using DESeq2^33^ R package as described in previous work^32^. Differentially expressed genes were defined “Upregulated” if adjusted P-value was lower than 0.05 and had log2(Fold Change)>1.5. Differentially expressed genes from RNA-seq data were enriched for functional gene categories using enrichr function from GSEApy^34^ python package. GO enrichment analysis was done against Biological processes, Cellular component and Molecular function datasets. Transcription factor analysis was done against ENCODE and ChEA Consensus TFs from ChIP-X database.

ATAC-seq of cortical neurons

Cortical neuron isolation and ATAC sequencing was performed according to the protocol of Eremenko et. al., 2021 ^35^. 3 samples of ATAC-seq data were prepared per each genotype. nextflow atacseq pipeline was for reads assembly. Differential peak analysis was conducted using Deseq2 with a particular summit interval. A peak was recognized as presented within a particular peak-summit interval if it can be found in at least 2 of 3 samples of a particular genotype. On the contrary, a peak was recognized as absent within a particular peak-summit interval if it can be found in less than 2 samples of a particular genotype. The obtained groups of emerging and vanishing peaks were annotated using R package annotatr (Cavalcante and Sartor 2017). Random peak intervals were retrieved using a function randomize_regions multiple times (random seeds are different). We retrieved randomized regions 10 times to construct an expected distribution of peak counts within a particular annotation. Assuming that the observed value of peak counts has the same standard deviation as the expected one, one-sided z-test was applied to test if the observed value significantly differs from the expected one. Differentially accessed genes from ATAC-seq data were analyzed using clusterProfiler^36^ R package.

Analysis of public REST and EZH2 ChIP-seq data

Processed data of REST ChIP-seq data from SHSY-5Y cells was used from previous published work^37^. Processed data of two human REST ChIP-seq replicated in human embryonic stem cells (hESC) (SRX3010118 and SRX3010119 accession numbers^38^) and two EZH2 ChIP-seq replicates embryonic stem cells (hESC) (SRX10382398 and SRX10382440 accession numbers^39^) were downloaded from ChIP-Atlas database^40^. Called peaks with q < 1 × 10^-5^ were annotated by their genome position using ‘annotatePeak’ function from ChIPseeker R package^41^ and only peaks localized between -3000 to 3000 bp around gene transcription start site (TSS) in all replicates were analyzed. The KEGG and Biological Process GO analysis of genes associated with the selected REST and EZH2 peaks was performed using ClusterProfiler. Enrichment analysis of overlapping genes was conducted using the enrichr function from GSEApy^34^ python package.

Public human brain microarray data

Transcription profile of SIRT6, SMAD4, SUZ12 and REST from 6 human brains was downloaded from Allen Brian Atlas – human microarray database^42^.

Generation of SIRT6 KO cells

The SIRT6 KO SHSY-5Y cell line used was generated by Kaluski et al^31^. Briefly, SH-SY-5Y cells were infected with lentivirus GeCKO system. We used 2sgRNA targeting Sirt6 CRISPR2 GCTGTCGCCGTACGCGGACA and CRISPR3 GCTCCACGGGAACATGTTTG and empty shRNA as a control. Constructions were kindly donated by the Aharoni Lab, Weitzmann Institute. Cells were selected by 2ug/ml puro for a week, followed by serial dilutions to single cell colony.

RNA extraction

RNA extraction from SHSY-5Y WT and SIRT6-KO cells was performed using the EZ-RNA II total RNA isolation kit from Biological Industries according to manufacturer’s protocol.

cDNA production

Isolated RNA (1µg per sample) was reverse transcribed to cDNA using qScript cDNA Synthesis Kit from Quantabio, according to manufacturer’s protocol.

qPCR

Quantitative qPCR was performed using ROCHE LightCycler® 480Probes Master or Bio -Rad SsoAdvanced Universal SYBR® Green Supermix, according to manufacturer’s protocol. qPCR data analysis was performed using -ΔΔCt method. Beta-Actin was used as housekeeping gene.

Chromatin extraction

Cells were collected and washed in PBS and resuspended in 2-5 pellet volumes of lysis buffer (10mM HEPES pH 7.4, 10mM KCl, 0.05% NP-40 and protease, deacetylase and phosphatase inhibitors). For tissue samples, brains were homogenized with lysis buffer in a bullet homogenizer. Samples were incubated 20 minutes on ice and centrifuged at 14,000 rpm at 4°C for 10 minutes. The supernatants containing the cytoplasmic proteins were removed and kept in a separate tube. Cell pellets were resuspended with 2-5 volumes of 0.2N HCl and incubated 20 minutes on ice, then centrifuged at 14,000 rpm at 4°C for 10 minutes. Supernatants were neutralized with an equal volume of 1M Tris-HCl pH 8.

Total protein extraction

For total protein extraction, cells were harvested via scraping and lysed with 1 ml of lysis buffer (KCl 150mM, Tris HCl pH 7.5 25mM, Glycerol 5%, Triton 0.1%, EDTA 0.2mM, PMSF 0.2mM, DDT 1mM, phosphatase inhibitor 1X), incubated on ice for 30 minutes, then centrifuged 14000 rpm 4°C 30 minutes. Supernatant was collected.

Western Blots and Immunostaining

Western blot was performed by loading protein samples (samples prepared with 4X Lameli and β-mercaptoethanol) to 6%-12% acryl amid gels. After electrophoresis, proteins were transferred to a nitrocellulose membrane. Prior to immunostaining, membranes were blocked using 5% skim milk or 5% BSA. After blocking, membranes were incubated with primary antibody (1:1000) overnight. Membranes were washed 10 minutes and 3 times in TTBS 1X. Secondary antibody (1:10000) was applied for 1hr in room temperature. 3 more washes were conducted before membrane was revealed.

DNA transfection

DNA transfection of recombinant DNA to cell culture was performed according to PolyJet™ DNA In Vitro Transfection Reagent manufacturer’s protocol. Cells were transfected with recombinant DNA (REST-WT, K494A, K494M, K494Q, SIRT6, CMV-Flag) 48h prior to sample collection.

REST expression in AD patients

REST expression values in AD patients were obtained from AD microarray data (GSE48350)^43–48^. Data was separated according to Braak stage.

ChIP-qPCR

ChIP–qPCR analysis ChIP-seq sample preparation was carried out as previously described^49^. Briefly, SHSY-5Y cells WT and SIRT6 KO, or WT and SIRT KO transfected with Flag-REST, K494A, K494M, K494Q were cultured up to 80% of confluence. In the case of ChIP negative controls cells were transfected with an empty vector (CMV-Flag). Cells were cross-linked with 1% formaldehyde for 10 minutes and blocked with 0.125M Glycine for 5 minutes. Cross-linked cells were scrapped on RIPA buffer (150mM NaCl, 1% NP-40, 0.5% Sodium deoxycholate, 0.1% SDS, 50mM Tris pH 8, 5mM EDTA, 0.5mM PMSF, 50mM NaF/0.2mM Sodium orthovanadate, 5uM trichostatin A), and chromatin was sonicated with a needle sonicator (VibraCell VCX130, Sonics, CT, USA) during 25 minutes, 30/30 seconds on/off cycles at 40% of amplitude. One milligram of the sonicated chromatin was incubated overnight with previously blocked SureBeads™ Protein G Magnetic Beads or Anti-FLAG® M2 Magnetic Beads. The beads were then washed 4 times with RIPA buffer, 4 times with LiCl buffer (500mM LiCl, 100mM Tris-HCl pH 8.5, 1% NP-40, 1% sodium deoxycholate), and 2 times with TE buffer (10mM Tris-HCl pH 8, 1mM EDTA). Chromatin was eluted from beads using elution buffer (70mM Tris-HCl pH 8, 1mM EDTA, 1.5% SDS) and cross-linking was reversed by adding 200mM NaCl, 1U of Proteinase K, and incubating the eluted chromatin at 65C for 5 hours. The obtained DNA was further purified using the Nucleo Spin Gel and PCR Clean-Up kit. For qPCR analysis of specific REST binding regions in the genome, we used the set of primers listed in the key resources table. qPCR was performed according to the method described. We used 1uL of Input and ChIP samples per qPCR reaction.

ChIP-seq

Endogenous REST and transfected REST samples were obtained using the same protocol for generating ChIP-qPCR samples. Eluted DNA was sent for sequencing and analysis at Azenta Life Sciences.

ChIP-Seq Library Preparation and Sequencing

ChIP DNA samples were quantified using Qubit 2.0 Fluorometer (Life Technologies, Carlsbad, CA, USA) and the DNA integrity was checked with 4200 TapeStation (Agilent Technologies, Palo Alto, CA, USA). ChIP-Seq library preparation and sequencing reactions were conducted at GENEWIZ, Inc/Azenta US, Inc. (South Plainfield, NJ, USA). NEB Next Ultra II DNA Library Preparation kit was used following the manufacturer’s recommendations (Illumina, San Diego, CA, USA). Briefly, the ChIP DNA was end repaired and adapters were ligated after adenylation of the 3’ends. Adapter-ligated DNA was size selected, followed by clean up, and limited cycle PCR enrichment. The ChIP library was validated using Agilent TapeStation and quantified using Qubit 2.0 Fluorometer as well as real time PCR (Applied Biosystems, Carlsbad, CA, USA). The sequencing libraries were multiplexed and clustered on one lane of a flowcell. After clustering, the flowcell was loaded on the Illumina Novaseq instrument according to manufacturer’s instructions (Illumina, San Diego, CA, USA). Sequencing was performed using a 2x150 Paired End (PE) configuration. Image analysis and base calling were conducted by the Novaseq Control Software (NCS). Raw sequence data (.bcl files) generated from Illumina instrument was converted into fastq files and demultiplexed using Illumina's bcl2fastq 2.17 software. One mis-match was allowed for index sequence identification.

ChIP-seq data analysis in SHSY-5Y cells

Raw fastq files from both experiments were analyzed with nf-core/chipseq pipeline (v2.0)^50^ . In brief, reads were trimmed with TrimGalore! ^51^ and align to the hg38 reference genome using BWA algorithm ^52^. The peak calling step was performed with MACS2 software ^53^, followed by the identification of the consensus and condition-specific peak sets. Peak annotation and enrichment analysis were performed with clusterProfiler R package ^54^.

Publicly available Lamin B ChIP-seq human brain data ^55^ was used to annotate LAD regions. Heterochromatin regions in SK-N-SH cells, defined using H3K27me3 and H3K9me3 peaks were downloaded from The Human Heterochromatin Chromatin Database (HHCDB) ^56^. The significance of the overlap between peak sets used as a measure for REST occurrence at LAD or heterochromatin regions was calculated with regioneR package ^57^ with ‘randomizeRegions’ argument in permTest() function, aiming to preserve the size of tested regions for permutations (N = 3000).Immunofluorescence

SH-SY5Y cells were washed with PBS and fixed with 4% paraformaldehyde for 10 minutes at RT, followed by two additional washes. Cells were permeabilized (0.1% NaCitrate, 0.1% Triton X-100, pH 6, in DDW) for 5 minutes and washed again. After 30 minutes blocking (0.5% BSA, 5% goat serum, 0.1% Tween-20 in PBS), cells were incubated with primary antibody (REST-1:1000, LAMINB-1:600, EZH2-1:500, Flag-1:900) diluted in blocking buffer overnight at 4°C. The next day, cells were washed three times with wash buffer (0.25% BSA, 0.1% Tween-20 in PBS), incubated for 1h with secondary antibody (diluted in blocking buffer 1:200) at RT and washed three more times. Cells were then DAPI-stained for three minutes at RT and washed with PBS twice before imaging.

Aggregate quantification using “AggreCount” tool

REST aggregates were quantified using ImageJ plugin “AggreCount”^58^. The parameters used for aggregate quantification are: Lower 15000.000. Upper 65535.000. perinuclear distance 10.000. aggresome size 4.000. minimum aggregate size 0.200. maximum aggregate size 20.000. minimum nuclei size 40.000. nuclei strictness 10.000. minimum cell size 75.000. cell strictness 10.000. nuclei channel 2.000. nuclei stack 1.000. nuclei frame 1.000. aggregate channel 1.000. aggregate stack 1.000. aggregate frame 1.000. cell channel 1.000. cell stack 1.000. cell frame 1.000. Find cells? 1.000. Segmentation? NaN. Batch mode 1.000.

REST "ring” positive cell quantification

Immunofluorescence was performed on SHSY-5Y cells (WT and KO) transfected with recombinant Flag-REST. Cells positive to the transfection were qualitatively evaluated for the appearance of the “REST ring” appearance, where ring positive cells scored with 1, and ring negative cells scored with 0. Percentage of “REST ring” cells was determined as the proportion of ring positive cell to the number of the total tranfected cells.

REST ring quantification

Immunofluorescence was performed on SHSY-5Y cells (WT and KO) transfected with recombinant Flag-REST. Transfected REST was identified using Anti-Flag primary antibody. Anti-LAMINB primary antibody and DAPI staining were used as standard signals for laminar and homogenous signal distribution, respectively. For each Flag-REST positive cell cross section, a signal distribution plot was measured for each channel, i.e. Flag (REST), LAMINB and DAPI. The intensity values along the cross section and length value of the cross-section were normalized to a unit value of 1 to remove intensity and length dimensions. For each channel, an area under the graph was calculated. REST Laminar co-localization value was measured as:

$$Laminar co-localization={log}_{10} (\frac{\left| red area-blue area \right|}{\left| red area-green area \right|})$$

while “red area” represents Flag-REST signal area value, “green area” represents Lamin B signal area value, and “blue area” represents DAPI signal area value. The closer REST localizes to Lamin B, the higher the REST Laminar co-localization value.

shSIRT6 cells treatment

shSRIT6 and shCtrl (scramble) cells were treated for 21, 10, 5 and 0 days with targeting SIRT6, and an empty shRNA as a control. Cells were selected by 2 μg/mL puromycin for a week.

Immunoprecipitation

Flag tagged REST was transfected to SHSY-5Y WT and KO cells. After 48 hr transfection cells were collected, harvested in 1ml Lysis buffer (KCl 0.5M, Tris HCl ph 7.5 50mM, NP40 1%, DTT 0.5mM, PMSF 0.2mM, phosphatase inhibitor 1X) and incubated on ice for 30 minutes, followed by centrifugation 14000 rpm, 30 minutes, 4°C. Protein samples were normalized to equal protein quantity, and incubated with M2 - Flag magnetic beads (Sigma M8823) for 2 hr in rotation, 4°C. Beads were washed 3 times in Lysis buffer, followed by 1 wash in SDAC buffer (Tris HCl pH 9 50mM, MgCl_2_ 4mM, NaCl 50mM, DTT 0.5 mM, PMSF 0.2mM, phosphatase inhibitor 1X). Flag-bound proteins were eluted using Flag peptide for 1 hr in rotation, 4°C.

Co-Immunoprecipitation

Flag-tagged REST was transfected to SHSY-5Y WT and KO cells. After 48 hr transfection cells were collected, harvested in 1ml Lysis buffer (KCl 150mM, Tris HCl pH 7.5 25mM, Glycerol 5%, Triton 0.1%, EDTA 0.2mM, PMSF 0.2mM, DDT 1mM, phosphatase inhibitor 1X) and incubated on ice for 30 minutes, followed by centrifugation 14000 rpm, 30 minutes, 4°C. Protein samples were normalized to equal protein quantity, and incubated with M2 - Flag magnetic beads (Sigma M8823) for 2 hr in rotation, 4°C. Beads were washed 2 times with Lysis buffer (KCl 150mM), followed by 2 washes of Lysis buffer (KCl 300mM) and one wash of Lysis buffer (KCl 150mM). Flag-bound proteins were eluted using Flag peptide for 1 hr in rotation, 4°C.

REST IP Mass spectrometry

For preparation of REST samples for mass spectrometry, Flag-REST was over expressed in WT and SIRT6 KO HEK293T cells. Flag-REST was immuno-precipitated according to the protocol previously described. IP samples were separated by SDS gel electrophoresis. Sample were sent for mass spectrometry and analysis at the Smoler Proteomics center at the Technion. Proteomics results were analyzed using R, code provided.

Quantification and statistical analysis

Pearson correlation test was performed using corr and personr funcitions in pandas^59^ and SciPy^60^ python packages, respectively. Hypergeometric test for 2 group Venn diagrams was performed using hypergeom.pmf function in SciPy python package. Permutation test for 3 group Venn diagram was performed by calculating the probability of randomly overlapping 3 groups of genes in the length of the measured groups from the background of human protein coding genes^61^, relative to the observed overlap, repeated 10000 times. Additional statistical analysis was performed using GraphPad Prism version 10.0.0, Boston, Massachusetts USA, www.graphpad.com, and included either t-test, one way ANOVA or 2-way ANOVA followed by post hoc Dunnet, Sidak or Tukey test. Statistical significance was determined when P-value was below 0.05 (detailed statistical analysis per experiment in Table S6).
